# Supplementary material for: Characterizing Healthy Dietary Practices in Japan: Insights from a 2024 Nationwide Survey and Cluster Analysis
Source: Nutrients. 2024 May 8;16(10):1412. doi: 10.3390/nu16101412 (PMC11124242; doi:10.3390/nu16101412)
Supplement: Supplementary file 1 [file nutrients-16-01412-s001.zip › nutrients-2983530-supplementary.pdf]

Supplementary text S1: Dimension reduction via Uniform Manifold Approximation and Projection (UMAP)

McInnes et al. (2018) introduced Uniform Manifold Approximation and Projection (UMAP),[30] a novel approach within the spectrum of unsupervised manifold learning methodologies aimed at dimensionality reduction. This method is grounded in the principles of Riemannian geometry and algebraic topology. When juxtaposed with prevalent dimension reduction techniques such as t-Distributed Stochastic Neighbour Embedding (t-SNE) and Principal Component Analysis (PCA), UMAP is distinguished by its enhanced computational efficiency and superior maintenance of the global structure of data in reduced dimensional spaces.

The UMAP algorithm encompasses a two-stage process: initially, it constructs a k-neighbourhood graph based on fuzzy set in original data space to depict the dataset's topology; subsequently, it undertakes the optimization of a cost function to generate a k-neighbourhood graph in lower dimensions that can closely resemble the original. In k-neighbourhood graphs, nodes symbolize data points, while edges denote the proximity between points, calculated using a distance metric appropriate for the manifold. The comprehensive algorithms are elaborated in McInnes et al. (2018),[30] with the uwot package in R facilitating the implementation of UMAP through various functions. In the context of this study, we projected ten binary variables and eight 7-point Likert scale variables onto a two-dimensional space (manifold) for subsequent visual analysis. Given the binary nature and Likert scale of the variables, we used the Hamming and Euclidean distances as the metrics for calculating manifold distances, respectively. The neighbourhood identification on the graph was performed with k set to 30, while other parameters adhered to the default settings of the uwot package.

Supplementary text S2: Comparative analysis of dietary patterns among clusters

For Outcome 1, we conducted inter-cluster comparisons, designating Cluster 4 (characterized by a lack of specific daily dietary patterns) as the reference group. This paper highlights the comparison with Cluster 3 (individuals who consume a diverse range of foods daily), identifying several statistically significant differences that can be largely attributed to distinct characteristics. Individuals in Cluster 3 were, on average, older, predominantly female, more highly educated, more likely to be married, less likely to smoke, and more likely to have a history of medical conditions. No significant differences were observed in residential location (Supplementary Table S2).

Moreover, Cluster 3 had a higher proportion of individuals with good well-being (24.3% vs. 14.1%) and a lower prevalence of social isolation (59.9% vs. 73.6%). Regarding dietary habits, Cluster 3 rated the importance of all 19 factors assessed in a survey about food selection criteria higher on a Likert scale than Cluster 4. Similarly, excluding digital environments and modern ordering systems, Cluster 3 respondents also placed higher importance on all 15 factors related to dining outside the home. Notably, individuals in Cluster 3 were more likely to indulge in snacking (41.7% vs. 24.1% reported snacking at least once daily) and consuming 'treat foods' under specific conditions more frequently (31.9% vs. 24.7% reported this behaviour as common or occasional). In terms of dining experiences, Cluster 3 reported having more time available for meals, with fewer individuals describing their time as 'not very sufficient' or 'not sufficient at all' compared to Cluster 4. Statistical differences were also observed in meal types, with a higher proportion of Cluster 3 engaging in home cooking and consuming frozen meals (42.1% vs. 37.8% reported eating frozen meals at least once a week). Additionally, within Cluster 3, there was a notably lower occurrence of individuals engaging in solitary dining or meals without conversation across all meals—breakfast, lunch, and dinner.

The use of food-related technology and applications saw notable differences between groups following the COVID-19 pandemic, with Cluster 3 exhibiting a higher engagement. Specifically, the adoption of recipe suggestion applications was significantly higher in Cluster 3 (24.0%) compared to Cluster 4 (12.3%). Additionally, the use of mapping applications for food-related purposes in Cluster 3 (23.0%) outpaced that in Cluster 4 (15.7%), indicating a stronger inclination towards locating food resources or dining options. Furthermore, applications dedicated to culinary skill enhancement and learning new recipes were more popular in Cluster 3 (9.7%) versus Cluster 4 (5.6%), suggesting a greater interest in cooking and recipe exploration within Cluster 3. Additionally, the frequency of using applications for making restaurant reservations in Cluster 3 (20.4%) was approximately 50% higher compared to Cluster 4 (13.2%).

Post-pandemic shifts in food-related consciousness were also more pronounced in Cluster 3. There was a marked increase in the stocking of long-lasting food items, such as canned and frozen goods, with 16.6% of Cluster 3 adopting this practice compared to only 9.4% in Cluster 4. Moreover, the

interest in recipes that reduce cooking time and in easy-to-prepare foods saw a greater upsurge in Cluster 3 (10.2%) than in Cluster 4 (6.6%), reflecting a shift towards more convenient cooking solutions amidst the pandemic's influence.

In the analysis for Outcome 2, Cluster D, which is characterized by a general lack of concern for the overall importance of dietary habits, served as the reference group for comparisons with other clusters. This paper focuses particularly on the comparison with Cluster C, where individuals perceive their diet to be of overall importance. A variety of statistically significant differences were identified, which can primarily be explained by the following unique characteristics of Cluster C: members tend to be older, predominantly female, have a higher BMI, more likely to be married, less likely to smoke, and more likely to have a history of medical conditions (Supplementary Table S3).

Cluster C also had a higher proportion of individuals reporting good well-being (23.1% vs. 16.6%) and fewer reports of social isolation (59.6% vs. 71.3%). In terms of dietary habits, Cluster C scored higher on the importance of nearly all 19 factors assessed in the survey on food selection criteria compared to Cluster D. Likewise, Cluster C respondents rated the importance of all 15 factors related to dining outside the home higher, with notable emphasis on snacking (40.2% vs. 30.0% reported snacking at least once daily) and consuming 'treat foods' under specific conditions more frequently (33.6% vs. 21.0% reported this behaviour as common or occasional). Regarding meal experiences, Cluster C reported greater temporal flexibility for meals, with fewer individuals finding their time to be 'not very sufficient' or 'not sufficient at all' compared to Cluster D. Moreover, it was observed that individuals in Cluster C were significantly less likely to partake in solitary eating or meals without engaging in conversation. There were statistical differences in meal types, with a higher frequency of home cooking and consumption of frozen meals among Cluster C (41.5% vs. 37.8% reported consuming frozen meals at least once a week).

The adoption of food-related technologies or applications was markedly more pronounced in Cluster C than in Cluster D. We revealed that Cluster C had significantly higher engagement with technology for food-related activities: 24.6% of Cluster C used recipe suggestion applications compared to 13.4% in Cluster D, and the use of map applications for locating food resources was equally higher in Cluster C (24.6%) versus Cluster D (13.6%). Furthermore, applications for enhancing cooking skills or learning new recipes were more frequently used in Cluster C (9.8%) as opposed to Cluster D (4.5%), and the disparity extended to restaurant reservation applications, with Cluster C's usage (21.6%) nearly doubling that of Cluster D (11.6%).

In terms of changes in awareness due to the COVID-19 pandemic, Cluster C showed a more significant adjustment in their food storage and preparation habits. There was a notable increase in the stockpiling of long-lasting food items, such as canned and frozen goods, with 16.6% of Cluster C

engaging in this practice compared to only 9.4% in Cluster D. Additionally, Cluster C exhibited a greater increase in interest towards recipes that reduce cooking time and easy-to-prepare foods, with 10.2% of its members seeking such solutions compared to 6.6% in Cluster D.

Supplementary Figure S1: Reachability plots for Outcomes 1 and 2, generated by OPTICS algorithm in clustering reduced-dimensional data

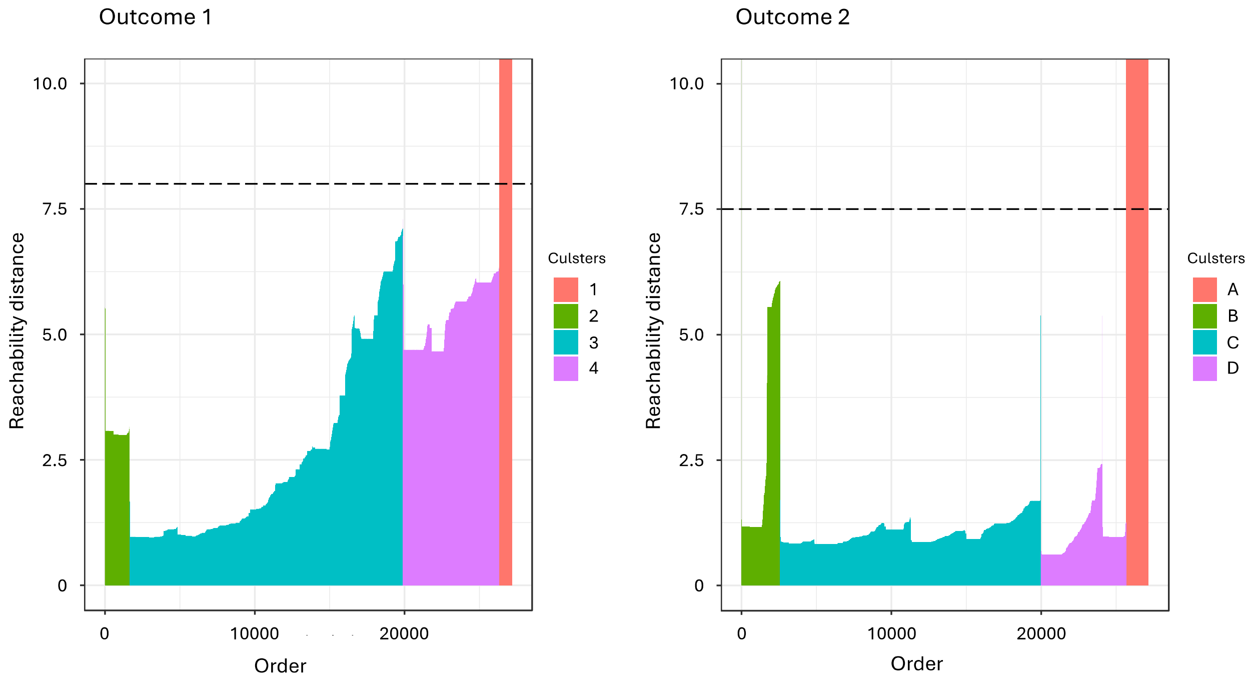

Outcome 1 refers to the Dietary Variety Score (DVS), which assesses dietary diversity based on responses to a questionnaire with ten items. These items ask about the frequency of consumption of ten specific food groups—seafood, soy products, green and yellow vegetables, meats, eggs, fats and oils, seaweeds, tubers, fruits, and milk—over the past week. The scoring system is binary: daily consumption is awarded one point, while all other frequencies score zero, resulting in a binary score for each of the ten food groups.

Outcome 2 refers to an indicator that measures the level of importance regarding nutritional and health aspects through a questionnaire comprising eight items. This measurement uses a seven-point Likert scale, ranging from 'not at all important' to 'extremely important', to evaluate the importance placed on nutritional and health factors when selecting meals and food products. The eight dietary and nutritional aspects covered include the reduction of salt, reduction of sugar, reduction of artificial additives, reduction of saturated fats, reduction of calories, increase of vitamins, increase of dietary fiber, and increase of unsaturated fats.

Supplementary Table S1: Results of the survey on items other than socio-demographic characteristics section

Variables	Number of respondents (%)
**II. Life satisfaction and social connections**	
Good Well-being	5777 (21.27)
Social Isolation	17328 (63.81)
**III. Habitual dietary preferences and tendencies**	
Frequency Level of Drinking Beverages	
Japanese tea (SA)	
Less than once per week	7497 (27.61)
1 to 2 times per week	3043 (11.21)
3 to 4 times per week	2237 (8.24)
5 to 6 times per week	1945 (7.16)
One serving daily	3994 (14.71)
2 to 3 servings daily	5528 (20.36)
4 to 6 servings daily	2088 (7.69)
7 to 9 servings daily	455 (1.68)
More than 10 servings daily	367 (1.35)
Oolong tea (SA)	
Less than once per week	21405 (78.83)
1 to 2 times per week	2395 (8.82)
3 to 4 times per week	1134 (4.18)
5 to 6 times per week	641 (2.36)
One serving daily	752 (2.77)
2 to 3 servings daily	488 (1.80)
4 to 6 servings daily	178 (0.66)
7 to 9 servings daily	51 (0.19)
More than 10 servings daily	110 (0.41)
Black tea (SA)	
Less than once per week	18331 (67.51)
1 to 2 times per week	3694 (13.60)
3 to 4 times per week	1809 (6.66)
5 to 6 times per week	944 (3.48)
One serving daily	1486 (5.47)
2 to 3 servings daily	597 (2.20)

4 to 6 servings daily	137 (0.50)
7 to 9 servings daily	50 (0.18)
More than 10 servings daily	106 (0.39)
Coffee (excluding canned coffee) (SA)	
Less than once per week	6410 (23.61)
1 to 2 times per week	2244 (8.26)
3 to 4 times per week	1909 (7.03)
5 to 6 times per week	2171 (8.00)
One serving daily	6208 (22.86)
2 to 3 servings daily	6450 (23.75)
4 to 6 servings daily	1262 (4.65)
7 to 9 servings daily	269 (0.99)
More than 10 servings daily	231 (0.85)
Canned coffee (SA)	
Less than once per week	21547 (79.35)
1 to 2 times per week	2294 (8.45)
3 to 4 times per week	1139 (4.19)
5 to 6 times per week	675 (2.49)
One serving daily	943 (3.47)
2 to 3 servings daily	349 (1.29)
4 to 6 servings daily	68 (0.25)
7 to 9 servings daily	38 (0.14)
More than 10 servings daily	101 (0.37)
Importance of Choices in Meals, Ingredients, and Groceries, mean (standard deviation) [min 0, max 7]	
Convenience in daily life (e.g., quick cooking, no hassle)	4.59 (1.24)
Preference in taste (e.g., strong, light, spicy)	4.85 (1.24)
Familiarity due to family or culture (e.g., eaten since childhood, family's traditional dishes)	4.15 (1.29)
Budget or price	5.13 (1.29)
Quality	5.02 (1.29)
Safety	5.24 (1.37)
Manufacturer or brand	3.99 (1.27)
Food allergies or physical constitution	3.88 (1.55)
Religion or beliefs	2.76 (1.52)

Medical considerations (restrictions due to illness or disability)	4.07 (1.46)
Origin of ingredients	4.30 (1.36)
Considerations of health and nutrition	4.80 (1.31)
Criteria related to best before and use-by dates	4.63 (1.29)
Freshness	5.15 (1.31)
Method of preservation (e.g., frozen, refrigerated, ambient)	4.57 (1.27)
Respect for culture and tradition	3.79 (1.34)
Impact on the environment (e.g., sustainability, organic)	3.91 (1.33)
Seasonality	4.52 (1.32)
Background information of ingredients and dishes (e.g., information about the producer)	3.96 (1.30)
Importance of the Scene or Environment for Eating Out, mean (standard deviation) [min 0, max 7]	
Environment conducive to enjoying conversation and communication	4.45 (1.30)
Consideration of the pace of dining (e.g., slow dining, fast service)	4.46 (1.25)
Sound environment (e.g., background noise, appropriate music)	4.18 (1.29)
Introduction of natural light	4.07 (1.32)
Appropriate lighting environment	4.32 (1.24)
Temperature setting (e.g., adjusted for the season)	4.59 (1.24)
Preferred location or seating (e.g., near a window or on a terrace)	4.22 (1.27)
Comfort of seating (e.g., chairs, height of tables)	4.49 (1.26)
Unique design or theme (e.g., scenery or culture)	3.72 (1.26)
Provision of privacy (e.g., private rooms or semi-private rooms)	4.32 (1.30)
Acceptance of all family members (e.g., with children or pets)	4.13 (1.42)
Digital environment (e.g., Wi-Fi, charging facilities)	3.87 (1.46)
Privacy of conversation	4.48 (1.30)
Modern ordering system (e.g., touch panels)	3.55 (1.30)
Visual information about ingredients and dishes (e.g., photos or descriptions)	4.11 (1.27)

Frequency of Snacking or Break Time (SA)	
More than twice a day	3058 (11.26)
Once a day	6915 (25.47)
4-6 times a week	2501 (9.21)
2-3 times a week	3896 (14.35)
About once a week	2021 (7.44)
1-2 times a month	1077 (3.97)
Rarely	7686 (28.31)
Main Time for Snacking or Break Time	
Morning (after breakfast until before lunch)	2940 (10.83)
After lunch until 3 PM	6228 (22.94)
3 PM until before dinner	10107 (37.22)
After dinner	4222 (15.55)
Late at night	963 (3.55)
No specific time	2118 (7.80)
Rarely	6728 (24.78)
Selection of Foods Normally Not Chosen in Specific Situations or Contexts (SA)	
Often	1192 (4.39)
Occasionally	6962 (25.64)
Rarely	7726 (28.45)
Almost never	11274 (41.52)
**IV. Everyday meal experiences**	
Meals Usually Prepared by Oneself	16039 (59.07)
Number of Days Eating Alone in the Past 7 Days	
Breakfast (SA)	
0 days	7821 (28.80)
1-2 days	2888 (10.64)
3-4 days	2075 (7.64)
5-6 days	2313 (8.52)
Every day (7 days)	8993 (33.12)
Usually do not eat breakfast	3064 (11.28)
Lunch (SA)	
0 days	5894 (21.71)
1-2 days	4429 (16.31)
3-4 days	3706 (13.65)

5-6 days	4379 (16.13)
Every day (7 days)	7349 (27.06)
Usually do not eat lunch	1397 (5.14)
Dinner (SA)	
0 days	12916 (47.57)
1-2 days	3363 (12.38)
3-4 days	2152 (7.93)
5-6 days	2167 (7.98)
Every day (7 days)	6002 (22.10)
Usually do not eat dinner	554 (2.04)
Frequently Experienced Conversation Situations	
Breakfast (SA)	
No conversation	13379 (49.27)
Simple conversation: Basic greetings or essential communication only	7422 (27.33)
Normal conversation: Everyday communication with friends or family	5947 (21.90)
Deep/long conversation: Detailed discussion on specific themes or topics	406 (1.50)
Lunch (SA)	
No conversation	11262 (41.47)
Simple conversation: Basic greetings or essential communication only	6678 (24.59)
Normal conversation: Everyday communication with friends or family	8698 (32.03)
Deep/long conversation: Detailed discussion on specific themes or topics	516 (1.90)
Dinner (SA)	
No conversation	6944 (25.57)
Simple conversation: Basic greetings or essential communication only	5376 (19.80)
Normal conversation: Everyday communication with friends or family	12625 (46.49)
Deep/long conversation: Detailed discussion on specific themes or topics	2209 (8.14)
Satisfaction with Meals	
Breakfast (SA)	
Not satisfied at all	912 (3.36)

Not very satisfied	1870 (6.89)
Neither satisfied nor dissatisfied	9460 (34.84)
Somewhat satisfied	11019 (40.58)
Very satisfied	3893 (14.34)
Lunch (SA)	
Not satisfied at all	661 (2.43)
Not very satisfied	1852 (6.82)
Neither satisfied nor dissatisfied	9468 (34.87)
Somewhat satisfied	11483 (42.29)
Very satisfied	3690 (13.59)
Dinner (SA)	
Not satisfied at all	555 (2.04)
Not very satisfied	1339 (4.93)
Neither satisfied nor dissatisfied	6634 (24.43)
Somewhat satisfied	12634 (46.53)
Very satisfied	5992 (22.07)
Perceived Time Availability During Meals	
Breakfast (SA)	
Not sufficient at all	1907 (7.02)
Not very sufficient	4724 (17.40)
Neither sufficient nor insufficient	6897 (25.40)
Somewhat sufficient	8521 (31.38)
Very sufficient	5105 (18.80)
Lunch (SA)	
Not sufficient at all	793 (2.92)
Not very sufficient	2597 (9.56)
Neither sufficient nor insufficient	7290 (26.85)
Somewhat sufficient	10558 (38.88)
Very sufficient	5916 (21.79)
Dinner (SA)	
Not sufficient at all	551 (2.03)
Not very sufficient	1442 (5.31)
Neither sufficient nor insufficient	4960 (18.27)
Somewhat sufficient	11122 (40.96)
Very sufficient	9079 (33.44)

Frequency of Meal Types	
Homemade meals at home (SA)	
Almost every day	15828 (58.29)
3-4 times a week	4420 (16.28)
1-2 times a week	2389 (8.80)
1-2 times a month	1152 (4.24)
Once or twice every six months	668 (2.46)
Rarely/Never	2697 (9.93)
Eating out (restaurants, fast food, etc.) (SA)	
Almost every day	351 (1.29)
3-4 times a week	911 (3.35)
1-2 times a week	4162 (15.33)
1-2 times a month	9598 (35.35)
Once or twice every six months	5839 (21.50)
Rarely/Never	6293 (23.18)
Takeout (bento, onigiri, etc.) (SA)	
Almost every day	631 (2.32)
3-4 times a week	1445 (5.32)
1-2 times a week	4039 (14.87)
1-2 times a month	6694 (24.65)
Once or twice every six months	4796 (17.66)
Rarely/Never	9549 (35.17)
Delivery services (SA)	
Almost every day	229 (0.84)
3-4 times a week	412 (1.52)
1-2 times a week	887 (3.27)
1-2 times a month	1551 (5.71)
Once or twice every six months	2556 (9.41)
Rarely/Never	21519 (79.25)
Eat-in at convenience stores or shopping malls (SA)	
Almost every day	296 (1.09)
3-4 times a week	708 (2.61)
1-2 times a week	1862 (6.86)
1-2 times a month	3921 (14.44)
Once or twice every six months	5297 (19.51)

Rarely/Never	15070 (55.50)
Prepared foods from supermarkets (SA)	
Almost every day	794 (2.92)
3-4 times a week	2588 (9.53)
1-2 times a week	8018 (29.53)
1-2 times a month	7516 (27.68)
Once or twice every six months	3141 (11.57)
Rarely/Never	5097 (18.77)
Light meals at cafes or tea shops (SA)	
Almost every day	239 (0.88)
3-4 times a week	494 (1.82)
1-2 times a week	1813 (6.68)
1-2 times a month	4643 (17.10)
Once or twice every six months	5843 (21.52)
Rarely/Never	14122 (52.01)
School or company cafeterias/canteens (SA)	
Almost every day	740 (2.73)
3-4 times a week	1161 (4.28)
1-2 times a week	1138 (4.19)
1-2 times a month	968 (3.56)
Once or twice every six months	943 (3.47)
Rarely/Never	22204 (81.77)
Instant food (SA)	
Almost every day	560 (2.06)
3-4 times a week	1583 (5.83)
1-2 times a week	6707 (24.70)
1-2 times a month	9008 (33.17)
Once or twice every six months	3968 (14.61)
Rarely/Never	5328 (19.62)
Frozen food (SA)	
Almost every day	824 (3.03)
3-4 times a week	2614 (9.63)
1-2 times a week	7665 (28.23)
1-2 times a month	7520 (27.69)
Once or twice every six months	3227 (11.88)

Rarely/Never	5304 (19.53)
Frequency of Methods for Purchasing Ingredients and Groceries	
Supermarket (SA)	
Almost every day	3193 (11.76)
3-4 times a week	7439 (27.40)
1-2 times a week	12087 (44.51)
1-2 times a month	2256 (8.31)
Once or twice every six months	637 (2.35)
Rarely/Never	1542 (5.68)
Convenience store (SA)	
Almost every day	601 (2.21)
3-4 times a week	1552 (5.72)
1-2 times a week	4384 (16.14)
1-2 times a month	6197 (22.82)
Once or twice every six months	4568 (16.82)
Rarely/Never	9852 (36.28)
Local market or direct sales outlet (SA)	
Almost every day	239 (0.88)
3-4 times a week	682 (2.51)
1-2 times a week	2484 (9.15)
1-2 times a month	4063 (14.96)
Once or twice every six months	4980 (18.34)
Rarely/Never	14706 (54.16)
Specialty stores (butcher, fishmonger, greengrocer, etc.) (SA)	
Almost every day	264 (0.97)
3-4 times a week	825 (3.04)
1-2 times a week	2783 (10.25)
1-2 times a month	3926 (14.46)
Once or twice every six months	3994 (14.71)
Rarely/Never	15362 (56.57)
Online shop (SA)	
Almost every day	175 (0.64)
3-4 times a week	374 (1.38)
1-2 times a week	1292 (4.76)

1-2 times a month	3136 (11.55)
Once or twice every six months	4111 (15.14)
Rarely/Never	18066 (66.53)
Meal kits (SA)	
Almost every day	182 (0.67)
3-4 times a week	418 (1.54)
1-2 times a week	927 (3.41)
1-2 times a month	1187 (4.37)
Once or twice every six months	1442 (5.31)
Rarely/Never	22998 (84.69)
Direct purchase from farmers or producers (SA)	
Almost every day	183 (0.67)
3-4 times a week	473 (1.74)
1-2 times a week	1528 (5.63)
1-2 times a month	2555 (9.41)
Once or twice every six months	3322 (12.23)
Rarely/Never	19093 (70.31)
Organic or pesticide-free specialty stores or online shops (SA)	
Almost every day	160 (0.59)
3-4 times a week	397 (1.46)
1-2 times a week	949 (3.49)
1-2 times a month	1267 (4.67)
Once or twice every six months	1689 (6.22)
Rarely/Never	22692 (83.57)
Large discount stores (SA)	
Almost every day	209 (0.77)
3-4 times a week	452 (1.66)
1-2 times a week	1311 (4.83)
1-2 times a month	2565 (9.45)
Once or twice every six months	3935 (14.49)
Rarely/Never	18682 (68.80)
Most Common Mode of Transportation for Grocery Shopping (SA)	
Walking	7246 (26.68)
Bicycle	4479 (16.49)

Self-driven car or motorcycle	12501 (46.04)
Car or motorcycle driven by someone else	2305 (8.49)
Bus, taxi, train	524 (1.93)
Other	99 (0.36)
Frequency of Participation in Social Dining or Food-centered Events	
Office or school lunch gatherings (SA)	
Almost every day	170 (0.63)
3-4 times a week	347 (1.28)
1-2 times a week	576 (2.12)
1-2 times a month	925 (3.41)
Once or twice every six months	1599 (5.89)
Rarely/Never	23537 (86.68)
Dining out with friends or colleagues (SA)	
Almost every day	145 (0.53)
3-4 times a week	391 (1.44)
1-2 times a week	1489 (5.48)
1-2 times a month	5252 (19.34)
Once or twice every six months	7956 (29.30)
Rarely/Never	11921 (43.90)
Special occasion meals with family or friends (birthdays, weddings, etc.) (SA)	
Almost every day	160 (0.59)
3-4 times a week	271 (1.00)
1-2 times a week	577 (2.12)
1-2 times a month	2116 (7.79)
Once or twice every six months	11467 (42.23)
Rarely/Never	12563 (46.27)
Outdoor dining like barbecues or picnics (SA)	
Almost every day	141 (0.52)
3-4 times a week	279 (1.03)
1-2 times a week	499 (1.84)
1-2 times a month	748 (2.75)
Once or twice every six months	4400 (16.20)
Rarely/Never	21087 (77.66)
Online meal gatherings (SA)	

Almost every day	159 (0.59)
3-4 times a week	285 (1.05)
1-2 times a week	468 (1.72)
1-2 times a month	624 (2.30)
Once or twice every six months	877 (3.23)
Rarely/Never	24741 (91.11)
Cooking classes or food workshops (SA)	
Almost every day	141 (0.52)
3-4 times a week	266 (0.98)
1-2 times a week	463 (1.71)
1-2 times a month	668 (2.46)
Once or twice every six months	932 (3.43)
Rarely/Never	24684 (90.90)
Casual meetings or study groups in cafes or tea shops (SA)	
Almost every day	158 (0.58)
3-4 times a week	275 (1.01)
1-2 times a week	636 (2.34)
1-2 times a month	1258 (4.63)
Once or twice every six months	2255 (8.30)
Rarely/Never	22572 (83.13)
Catered meals at events or parties (SA)	
Almost every day	137 (0.50)
3-4 times a week	273 (1.01)
1-2 times a week	453 (1.67)
1-2 times a month	707 (2.60)
Once or twice every six months	1898 (6.99)
Rarely/Never	23686 (87.23)
After-work events with meals or drinks (SA)	
Almost every day	136 (0.50)
3-4 times a week	301 (1.11)
1-2 times a week	682 (2.51)
1-2 times a month	1774 (6.53)
Once or twice every six months	4047 (14.90)
Rarely/Never	20214 (74.44)
Community or local food events (SA)	

Almost every day	143 (0.53)
3-4 times a week	264 (0.97)
1-2 times a week	507 (1.87)
1-2 times a month	783 (2.88)
Once or twice every six months	2329 (8.58)
Rarely/Never	23128 (85.17)
Use of Technology or Apps Related to Food and Ingredients	
Recipe suggestion apps (e.g., Cookpad, Delish Kitchen, etc.)	5646 (20.79)
Calorie and nutrition management apps (e.g., MyFitnessPal, AskKen, etc.)	865 (3.19)
Inventory and expiration date management apps for ingredients and groceries	570 (2.10)
Food waste prevention apps (e.g., notifications about ingredients and groceries nearing expiration, discounted food notifications)	626 (2.31)
Delivery service apps for ingredients (e.g., Oisix, Rakuten Mart, etc.)	738 (2.72)
Restaurant reservation and review apps (e.g., Tabelog, Retty, etc.)	4991 (18.38)
Apps providing information on the origin and sustainability of ingredients (e.g., CodeCheck, etc.)	486 (1.79)
Diet support apps for avoiding allergies or specific ingredients	403 (1.48)
Control and management apps for smart kitchen devices (e.g., IoT refrigerators, smart ovens, etc.)	483 (1.78)
AR (Augmented Reality) cooking support apps	265 (0.98)
Apps for purchasing regional specialties (e.g., selecting return gifts for Furusato Nozei, specialty product market apps, etc.)	1152 (4.24)
Apps for learning new recipes or improving cooking skills (e.g., Udemy, YouTube channels, etc.)	2289 (8.43)
Map apps (e.g., Google Maps, etc.)	5703 (21.00)
None apply	14756 (54.34)
Other	28 (0.10)
Changes in Your Food and Ingredients-Related Habits After the COVID-19 Pandemic	
Emphasizing safety and hygiene management in choosing ingredients and groceries	3205 (11.80)

Decrease in frequency of dining out and increase in frequency of cooking at home	5145 (18.95)
Increase in use of online shopping and delivery services for ingredients and groceries	1387 (5.11)
Stockpiling long-lasting food items (canned goods, frozen foods, etc.)	3943 (14.52)
Interest in home gardening or urban agriculture and starting to practice it	1361 (5.01)
Increased awareness of supporting and purchasing locally produced ingredients and groceries	2304 (8.48)
Concern about the hygiene management and crowding of restaurants when dining out	4069 (14.98)
Increased interest in ingredients and groceries or supplements that boost immunity	3250 (11.97)
Gathering and learning information about the supply chain and distribution of food and groceries	838 (3.09)
Increased participation and interest in online cooking classes and meal gatherings	420 (1.55)
Efforts to reduce food waste and increase awareness of reuse	3833 (14.12)
Preference for restaurants and cafes that introduce contactless payment and automated serving	956 (3.52)
Interest in recipes that shorten cooking time and simple cooking foods	2506 (9.23)
Spending more time and effort on cooking, trying new recipes, and authentic dishes	1370 (5.05)
None apply	13553 (49.91)
Other	27 (0.10)

SA refers to single-answer questions.

Supplementary Table S2: Results of the survey for Outcome 1 by clusters: number (%)

	Cluster 1	Cluster 2	Cluster 3	Cluster 4
	n=845, 3.11%	n=1641, 6.03%	n=18244, 67.19%	n=6424, 23.66%
Variables				
**I. Sociodemographic characteristics**				
Age, mean (standard deviation)	46.73 (15.61)	53.91 (15.48)	* 56.16 (16.33)	* 47.16 (15.77)
Gender (SA)				*
Female	393 (46.5)	708 (43.1)	9290 (50.9)	2612 (40.7)
Male	449 (53.1)	925 (56.4)	8856 (48.5)	3767 (58.6)
Other	3 (0.4)	8 (0.5)	98 (0.5)	45 (0.7)
Body-mass index (BMI), mean (standard deviation)	22.18 (3.29)	22.16 (3.13)	21.82 (3.13)	21.97 (3.27)
Residence (SA)				
Hokkaido	41 (4.9)	65 (4.0)	703 (3.9)	270 (4.2)
Tohoku				
Aomori	7 (0.8)	23 (1.4)	198 (1.1)	70 (1.1)
Iwate	4 (0.5)	13 (0.8)	156 (0.9)	65 (1.0)
Miyagi	9 (1.1)	26 (1.6)	437 (2.4)	126 (2.0)
Akita	5 (0.6)	14 (0.9)	169 (0.9)	49 (0.8)
Yamagata	8 (0.9)	11 (0.7)	165 (0.9)	59 (0.9)
Fukushima	18 (2.1)	21 (1.3)	191 (1.0)	75 (1.2)
Kanto				
Ibaraki	7 (0.8)	25 (1.5)	302 (1.7)	116 (1.8)
Tochigi	9 (1.1)	19 (1.2)	167 (0.9)	77 (1.2)
Gunma	9 (1.1)	19 (1.2)	195 (1.1)	86 (1.3)
Saitama	52 (6.2)	83 (5.1)	979 (5.4)	381 (5.9)
Chiba	45 (5.3)	86 (5.2)	897 (4.9)	307 (4.8)
Tokyo	104 (12.3)	208 (12.7)	2325 (12.7)	881 (13.7)
Kanagawa	57 (6.7)	119 (7.3)	1441 (7.9)	487 (7.6)
Chubu				
Niigata	12 (1.4)	23 (1.4)	290 (1.6)	83 (1.3)
Toyama	7 (0.8)	6 (0.4)	148 (0.8)	45 (0.7)
Ishikawa	10 (1.2)	11 (0.7)	171 (0.9)	53 (0.8)
Fukui	4 (0.5)	14 (0.9)	89 (0.5)	34 (0.5)

Yamanashi	9 (1.1)	13 (0.8)	102 (0.6)	28 (0.4)
Nagano	11 (1.3)	17 (1.0)	311 (1.7)	88 (1.4)
Gifu	13 (1.5)	22 (1.3)	310 (1.7)	112 (1.7)
Shizuoka	27 (3.2)	28 (1.7)	482 (2.6)	169 (2.6)
Aichi	63 (7.5)	110 (6.7)	1376 (7.5)	426 (6.6)
Kinki				
Mie	5 (0.6)	15 (0.9)	216 (1.2)	68 (1.1)
Shiga	4 (0.5)	17 (1.0)	166 (0.9)	68 (1.1)
Kyoto	13 (1.5)	38 (2.3)	371 (2.0)	109 (1.7)
Osaka	53 (6.3)	146 (8.9)	1230 (6.7)	480 (7.5)
Hyogo	34 (4.0)	95 (5.8)	814 (4.5)	231 (3.6)
Nara	3 (0.4)	17 (1.0)	171 (0.9)	65 (1.0)
Wakayama	9 (1.1)	16 (1.0)	110 (0.6)	32 (0.5)
Chugoku				
Tottori	3 (0.4)	9 (0.5)	82 (0.4)	25 (0.4)
Shimane	7 (0.8)	7 (0.4)	79 (0.4)	32 (0.5)
Okayama	11 (1.3)	29 (1.8)	307 (1.7)	89 (1.4)
Hiroshima	19 (2.2)	37 (2.3)	485 (2.7)	140 (2.2)
Yamaguchi	9 (1.1)	15 (0.9)	192 (1.1)	54 (0.8)
Shikoku				
Tokushima	5 (0.6)	13 (0.8)	102 (0.6)	43 (0.7)
Kagawa	11 (1.3)	21 (1.3)	149 (0.8)	57 (0.9)
Ehime	9 (1.1)	14 (0.9)	193 (1.1)	62 (1.0)
Kochi	4 (0.5)	6 (0.4)	79 (0.4)	29 (0.5)
Kyushu				
Fukuoka	54 (6.4)	82 (5.0)	908 (5.0)	326 (5.1)
Saga	3 (0.4)	8 (0.5)	92 (0.5)	51 (0.8)
Nagasaki	10 (1.2)	18 (1.1)	180 (1.0)	70 (1.1)
Kumamoto	14 (1.7)	13 (0.8)	186 (1.0)	73 (1.1)
Oita	10 (1.2)	12 (0.7)	125 (0.7)	58 (0.9)
Miyazaki	5 (0.6)	9 (0.5)	106 (0.6)	45 (0.7)
Kagoshima	6 (0.7)	14 (0.9)	169 (0.9)	73 (1.1)
Okinawa	13 (1.5)	14 (0.9)	128 (0.7)	57 (0.9)
Educational Background (SA)			*	
Junior high school graduate	26 (3.1)	29 (1.8)	374 (2.0)	172 (2.7)

High school / technical college graduate or enrolled	288 (34.1)	564 (34.4)	5839 (32.0)	2290 (35.6)
Junior college / vocational school graduate or enrolled	182 (21.5)	288 (17.6)	3511 (19.2)	1076 (16.7)
University graduate or enrolled	309 (36.6)	664 (40.5)	7683 (42.1)	2550 (39.7)
Graduate school completed or enrolled	40 (4.7)	67 (4.1)	815 (4.5)	257 (4.0)
Occupation (SA)	*	*	*	
Managerial occupation	39 (4.6)	105 (6.4)	1077 (5.9)	451 (7.0)
Professional or technical occupation	113 (13.4)	190 (11.6)	1949 (10.7)	768 (12.0)
Clerical worker	119 (14.1)	232 (14.1)	2212 (12.1)	941 (14.6)
Sales worker	57 (6.7)	80 (4.9)	783 (4.3)	378 (5.9)
Service worker	122 (14.4)	191 (11.6)	1654 (9.1)	831 (12.9)
Security worker	7 (0.8)	15 (0.9)	123 (0.7)	78 (1.2)
Agriculture, forestry, and fisheries worker	5 (0.6)	11 (0.7)	114 (0.6)	51 (0.8)
Production process worker	44 (5.2)	96 (5.9)	645 (3.5)	383 (6.0)
Transport and machinery operation worker	8 (0.9)	19 (1.2)	172 (0.9)	108 (1.7)
Construction and mining worker	6 (0.7)	18 (1.1)	187 (1.0)	116 (1.8)
Material moving, cleaning, packing, etc. worker	27 (3.2)	47 (2.9)	343 (1.9)	156 (2.4)
Student	18 (2.1)	18 (1.1)	232 (1.3)	167 (2.6)
Full-time homemaker	119 (14.1)	228 (13.9)	3623 (19.9)	633 (9.9)
Other (including unemployed, retired)	161 (19.1)	391 (23.8)	5130 (28.1)	1363 (21.2)
Annual Income (SA)		*	*	
Less than 2 million yen / about Less than $15,000	145 (17.2)	259 (15.8)	2943 (16.1)	1245 (19.4)
2 to under 4 million yen / about $15,400-$31,000	228 (27.0)	502 (30.6)	5424 (29.7)	1777 (27.7)
4 to under 6 million yen / about $30,800-$46,000	194 (23.0)	357 (21.8)	4062 (22.3)	1428 (22.2)
6 to under 8 million yen / about $46,200-$62,000	132 (15.6)	240 (14.6)	2592 (14.2)	887 (13.8)
8 to under 10 million yen / about $61,500-$77,000	78 (9.2)	137 (8.3)	1485 (8.1)	545 (8.5)

10 to under 20 million yen / about $76,900-$154,000	60 (7.1)	132 (8.0)	1438 (7.9)	428 (6.7)
Over 20 million yen / Over $154,000	8 (0.9)	14 (0.9)	300 (1.6)	114 (1.8)
Marital Status (SA)			*	*
Married (including common-law marriage)	467 (55.3)	999 (60.9)	11713 (64.2)	3191 (49.7)
Single (no partner)	234 (27.7)	359 (21.9)	3726 (20.4)	2143 (33.4)
Single (with a partner)	61 (7.2)	114 (6.9)	1059 (5.8)	628 (9.8)
Widowed	23 (2.7)	54 (3.3)	753 (4.1)	148 (2.3)
Divorced	60 (7.1)	121 (7.4)	1208 (6.6)	407 (6.3)
Smoking (SA)				*
Smokes daily	188 (22.2)	350 (21.3)	2925 (16.0)	1456 (22.7)
Smokes occasionally	12 (1.4)	31 (1.9)	286 (1.6)	125 (1.9)
Used to smoke but has not smoked for over a month	116 (13.7)	309 (18.8)	3440 (18.9)	1002 (15.6)
Does not smoke	529 (62.6)	951 (58.0)	11593 (63.5)	3841 (59.8)
Drinking (SA)				*
Daily	168 (19.9)	244 (14.9)	3094 (17.0)	989 (15.4)
5-6 days per week	42 (5.0)	82 (5.0)	713 (3.9)	324 (5.0)
3-4 days per week	47 (5.6)	113 (6.9)	1187 (6.5)	397 (6.2)
1-2 days per week	91 (10.8)	193 (11.8)	2113 (11.6)	678 (10.6)
1-3 days per month	79 (9.3)	159 (9.7)	1704 (9.3)	632 (9.8)
Rarely drinks	155 (18.3)	268 (16.3)	2895 (15.9)	978 (15.2)
Stopped drinking	18 (2.1)	65 (4.0)	645 (3.5)	192 (3.0)
Does not drink (cannot drink)	245 (29.0)	493 (30.0)	5348 (29.3)	2167 (33.7)
Health Condition (SA)			*	*
Good	116 (13.7)	192 (11.7)	3057 (16.8)	780 (12.1)
Fairly good	222 (26.3)	465 (28.3)	5261 (28.8)	1506 (23.4)
Average	363 (43.0)	708 (43.1)	7129 (39.1)	2871 (44.7)
Not very good	113 (13.4)	231 (14.1)	2315 (12.7)	934 (14.5)
Poor	31 (3.7)	45 (2.7)	482 (2.6)	333 (5.2)
Frequency of Device Use				
Wearable devices (SA)				*
Almost every day	51 (6.0)	99 (6.0)	1422 (7.8)	307 (4.8)
2-5 days per week	11 (1.3)	13 (0.8)	236 (1.3)	131 (2.0)

About once a week or less	15 (1.8)	25 (1.5)	292 (1.6)	142 (2.2)
Do not use	768 (90.9)	1491 (90.9)	16215 (88.9)	5852 (91.1)
IOT appliances (SA)				*
Almost every day	15 (1.8)	35 (2.1)	654 (3.6)	129 (2.0)
2-5 days per week	9 (1.1)	22 (1.3)	273 (1.5)	98 (1.5)
About once a week or less	13 (1.5)	25 (1.5)	247 (1.4)	214 (3.3)
Do not use	808 (95.6)	1560 (95.1)	16987 (93.1)	6051 (94.2)
Frequency of Social Media Use				
Facebook (SA)				*
Almost every day	73 (8.6)	122 (7.4)	1761 (9.7)	495 (7.7)
2-5 days per week	42 (5.0)	77 (4.7)	947 (5.2)	308 (4.8)
About once a week or less	90 (10.7)	189 (11.5)	1986 (10.9)	640 (10.0)
Do not use	640 (75.7)	1253 (76.4)	13550 (74.3)	4981 (77.5)
X/Twitter (SA)			*	*
Almost every day	243 (28.8)	328 (20.0)	4011 (22.0)	1696 (26.4)
2-5 days per week	75 (8.9)	121 (7.4)	1344 (7.4)	509 (7.9)
About once a week or less	87 (10.3)	167 (10.2)	1593 (8.7)	597 (9.3)
Do not use	440 (52.1)	1025 (62.5)	11296 (61.9)	3622 (56.4)
LINE (SA)		*		
Almost every day	507 (60.0)	877 (53.4)	9966 (54.6)	3344 (52.1)
2-5 days per week	125 (14.8)	255 (15.5)	2717 (14.9)	1012 (15.8)
About once a week or less	74 (8.8)	175 (10.7)	1911 (10.5)	687 (10.7)
Do not use	139 (16.4)	334 (20.4)	3650 (20.0)	1381 (21.5)
Instagram (SA)			*	*
Almost every day	233 (27.6)	308 (18.8)	3975 (21.8)	1575 (24.5)
2-5 days per week	70 (8.3)	111 (6.8)	1198 (6.6)	464 (7.2)
About once a week or less	78 (9.2)	156 (9.5)	1581 (8.7)	527 (8.2)
Do not use	464 (54.9)	1066 (65.0)	11490 (63.0)	3858 (60.1)
Youtube (SA)		*	*	*
Almost every day	390 (46.2)	556 (33.9)	6589 (36.1)	2593 (40.4)
2-5 days per week	147 (17.4)	301 (18.3)	3100 (17.0)	1100 (17.1)
About once a week or less	153 (18.1)	332 (20.2)	3622 (19.9)	1052 (16.4)
Do not use	155 (18.3)	452 (27.5)	4933 (27.0)	1679 (26.1)
Tiktok (SA)			*	*
Almost every day	101 (12.0)	122 (7.4)	1587 (8.7)	716 (11.1)

2-5 days per week	34 (4.0)		52 (3.2)		598 (3.3)		269 (4.2)
About once a week or less	44 (5.2)		79 (4.8)		898 (4.9)		373 (5.8)
Do not use	666 (78.8)		1388 (84.6)		15161 (83.1)		5066 (78.9)
Medical History							
Hypertension	117 (13.8)		309 (18.8)	*	3838 (21.0)	*	889 (13.8)
Diabetes	37 (4.4)		88 (5.4)		1262 (6.9)	*	300 (4.7)
Dyslipidemia (hyperlipidemia)	52 (6.2)		153 (9.3)	*	1976 (10.8)	*	331 (5.2)
Pneumonia / Bronchitis	27 (3.2)		55 (3.4)		714 (3.9)	*	164 (2.6)
Asthma	66 (7.8)	*	85 (5.2)		1057 (5.8)		311 (4.8)
Atopic dermatitis	72 (8.5)	*	76 (4.6)		948 (5.2)		290 (4.5)
Allergic rhinitis	97 (11.5)	*	143 (8.7)		1832 (10.0)	*	439 (6.8)
Periodontal disease	83 (9.8)		163 (9.9)		2315 (12.7)	*	472 (7.3)
Dental caries (cavities)	165 (19.5)	*	248 (15.1)		3755 (20.6)	*	818 (12.7)
Cataract	28 (3.3)		93 (5.7)	*	1549 (8.5)	*	212 (3.3)
Angina / Myocardial infarction	15 (1.8)		38 (2.3)		446 (2.4)	*	95 (1.5)
Stroke (cerebral infarction, cerebral hemorrhage)	7 (0.8)		17 (1.0)		258 (1.4)		55 (0.9)
COPD (Chronic Obstructive Pulmonary Disease)	5 (0.6)		5 (0.3)		74 (0.4)		16 (0.2)
Chronic kidney disease	5 (0.6)		9 (0.5)		149 (0.8)		40 (0.6)
Chronic hepatitis / Cirrhosis	6 (0.7)		5 (0.3)		113 (0.6)		29 (0.5)
Immunodeficiency or immune function decline (including those on steroids, biologics, immunosuppressants)	8 (0.9)		11 (0.7)		212 (1.2)	*	40 (0.6)
Cancer / Malignant tumor	29 (3.4)		83 (5.1)	*	1191 (6.5)	*	181 (2.8)
Chronic pain (e.g., persistent back pain, headache for over three months)	21 (2.5)		50 (3.0)		809 (4.4)	*	149 (2.3)
Depression	43 (5.1)		90 (5.5)		791 (4.3)		308 (4.8)
Mental illness other than depression	34 (4.0)		49 (3.0)		668 (3.7)		245 (3.8)
None apply	397 (47.0)	*	756 (46.1)	*	7046 (38.6)	*	3640 (56.7)
**II. Life satisfaction and social connections**							
Good Well-being	146 (17.3)		291 (17.7)	*	4435 (24.3)	*	905 (14.1)

Social Isolation	588 (69.6)	1085 (66.1)	*	10926 (59.9)	*	4729 (73.6)
**III. Habitual dietary preferences and tendencies**						
Frequency Level of Drinking Beverages						
Japanese tea (SA)			*		*	
Less than once per week	328 (38.8)	468 (28.5)		4403 (24.1)		2298 (35.8)
1 to 2 times per week	90 (10.7)	200 (12.2)		1874 (10.3)		879 (13.7)
3 to 4 times per week	58 (6.9)	149 (9.1)		1365 (7.5)		665 (10.4)
5 to 6 times per week	68 (8.0)	121 (7.4)		1235 (6.8)		521 (8.1)
One serving daily	102 (12.1)	268 (16.3)		2803 (15.4)		821 (12.8)
2 to 3 servings daily	137 (16.2)	292 (17.8)		4283 (23.5)		816 (12.7)
4 to 6 servings daily	45 (5.3)	108 (6.6)		1642 (9.0)		293 (4.6)
7 to 9 servings daily	9 (1.1)	22 (1.3)		360 (2.0)		64 (1.0)
More than 10 servings daily	8 (0.9)	13 (0.8)		279 (1.5)		67 (1.0)
Oolong tea (SA)			*		*	
Less than once per week	678 (80.2)	1311 (79.9)		14626 (80.2)		4790 (74.6)
1 to 2 times per week	72 (8.5)	148 (9.0)		1513 (8.3)		662 (10.3)
3 to 4 times per week	34 (4.0)	57 (3.5)		658 (3.6)		385 (6.0)
5 to 6 times per week	17 (2.0)	40 (2.4)		383 (2.1)		201 (3.1)
One serving daily	18 (2.1)	37 (2.3)		507 (2.8)		190 (3.0)
2 to 3 servings daily	18 (2.1)	29 (1.8)		335 (1.8)		106 (1.7)
4 to 6 servings daily	4 (0.5)	13 (0.8)		121 (0.7)		40 (0.6)
7 to 9 servings daily	1 (0.1)	1 (0.1)		33 (0.2)		16 (0.2)
More than 10 servings daily	3 (0.4)	5 (0.3)		68 (0.4)		34 (0.5)
Black tea (SA)					*	
Less than once per week	593 (70.2)	1187 (72.3)		12154 (66.6)		4397 (68.4)
1 to 2 times per week	114 (13.5)	195 (11.9)		2499 (13.7)		886 (13.8)
3 to 4 times per week	56 (6.6)	80 (4.9)		1209 (6.6)		464 (7.2)
5 to 6 times per week	31 (3.7)	50 (3.0)		625 (3.4)		238 (3.7)
One serving daily	33 (3.9)	82 (5.0)		1101 (6.0)		270 (4.2)
2 to 3 servings daily	15 (1.8)	31 (1.9)		451 (2.5)		100 (1.6)
4 to 6 servings daily	2 (0.2)	10 (0.6)		99 (0.5)		26 (0.4)
7 to 9 servings daily	0 (0.0)	1 (0.1)		39 (0.2)		10 (0.2)
More than 10 servings daily	1 (0.1)	5 (0.3)		67 (0.4)		33 (0.5)

Coffee (excluding canned coffee) (SA)		*		*		*	
Less than once per week	267 (31.6)		280 (17.1)		3736 (20.5)		2127 (33.1)
1 to 2 times per week	72 (8.5)		109 (6.6)		1311 (7.2)		752 (11.7)
3 to 4 times per week	66 (7.8)		119 (7.3)		1098 (6.0)		626 (9.7)
5 to 6 times per week	61 (7.2)		139 (8.5)		1319 (7.2)		652 (10.1)
One serving daily	159 (18.8)		442 (26.9)		4538 (24.9)		1069 (16.6)
2 to 3 servings daily	181 (21.4)		432 (26.3)		4931 (27.0)		906 (14.1)
4 to 6 servings daily	26 (3.1)		90 (5.5)		952 (5.2)		194 (3.0)
7 to 9 servings daily	8 (0.9)		15 (0.9)		200 (1.1)		46 (0.7)
More than 10 servings daily	5 (0.6)		15 (0.9)		159 (0.9)		52 (0.8)
Canned coffee (SA)				*		*	
Less than once per week	645 (76.3)		1267 (77.2)		14955 (82.0)		4680 (72.9)
1 to 2 times per week	67 (7.9)		167 (10.2)		1355 (7.4)		705 (11.0)
3 to 4 times per week	41 (4.9)		58 (3.5)		634 (3.5)		406 (6.3)
5 to 6 times per week	30 (3.6)		44 (2.7)		372 (2.0)		229 (3.6)
One serving daily	38 (4.5)		66 (4.0)		603 (3.3)		236 (3.7)
2 to 3 servings daily	21 (2.5)		25 (1.5)		205 (1.1)		98 (1.5)
4 to 6 servings daily	2 (0.2)		3 (0.2)		37 (0.2)		26 (0.4)
7 to 9 servings daily	0 (0.0)		5 (0.3)		21 (0.1)		12 (0.2)
More than 10 servings daily	1 (0.1)		6 (0.4)		62 (0.3)		32 (0.5)
Importance of Choices in Meals, Ingredients, and Groceries, mean (standard deviation) [min 0, max 7]							
Convenience in daily life (e.g., quick cooking, no hassle)	4.64 (1.28)	*	4.60 (1.14)	*	4.67 (1.21)	*	4.35 (1.30)
Preference in taste (e.g., strong, light, spicy)	4.87 (1.23)	*	4.76 (1.19)	*	4.97 (1.20)	*	4.52 (1.32)
Familiarity due to family or culture (e.g., eaten since childhood, family's traditional dishes)	3.99 (1.30)		4.08 (1.22)	*	4.24 (1.29)	*	3.91 (1.28)
Budget or price	5.26 (1.31)	*	5.09 (1.20)	*	5.24 (1.23)	*	4.81 (1.40)
Quality	4.79 (1.28)	*	4.93 (1.21)	*	5.20 (1.22)	*	4.54 (1.36)
Safety	4.99 (1.33)	*	5.15 (1.26)	*	5.44 (1.30)	*	4.70 (1.44)
Manufacturer or brand	3.83 (1.23)		3.98 (1.19)	*	4.06 (1.27)	*	3.83 (1.27)

Food allergies or physical constitution	3.78 (1.56)		3.87 (1.44)		3.92 (1.59)	*	3.78 (1.45)
Religion or beliefs	2.61 (1.49)	*	2.82 (1.48)		2.69 (1.54)	*	2.94 (1.48)
Medical considerations (restrictions due to illness or disability)	3.86 (1.42)		4.02 (1.37)	*	4.16 (1.49)	*	3.86 (1.37)
Origin of ingredients	4.00 (1.34)		4.20 (1.26)	*	4.45 (1.35)	*	3.96 (1.35)
Considerations of health and nutrition	4.47 (1.33)	*	4.62 (1.20)	*	5.02 (1.25)	*	4.28 (1.33)
Criteria related to best before and use-by dates	4.49 (1.26)		4.56 (1.20)	*	4.76 (1.27)	*	4.32 (1.34)
Freshness	4.91 (1.30)	*	5.06 (1.22)	*	5.36 (1.23)	*	4.61 (1.39)
Method of preservation (e.g., frozen, refrigerated, ambient)	4.41 (1.28)	*	4.51 (1.17)	*	4.71 (1.24)	*	4.21 (1.29)
Respect for culture and tradition	3.52 (1.35)		3.77 (1.27)	*	3.87 (1.35)	*	3.62 (1.31)
Impact on the environment (e.g., sustainability, organic)	3.53 (1.37)		3.80 (1.23)		4.03 (1.33)	*	3.66 (1.30)
Seasonality	4.15 (1.35)		4.39 (1.21)	*	4.71 (1.28)	*	4.05 (1.31)
Background information of ingredients and dishes (e.g., information about the producer)	3.59 (1.30)		3.90 (1.21)	*	4.05 (1.30)	*	3.75 (1.30)
Importance of the Scene or Environment for Eating Out, mean (standard deviation) [min 0, max 7]							
Environment conducive to enjoying conversation and communication	4.29 (1.37)		4.38 (1.19)	*	4.58 (1.28)	*	4.12 (1.32)
Consideration of the pace of dining (e.g., slow dining, fast service)	4.32 (1.29)		4.41 (1.16)	*	4.58 (1.23)	*	4.15 (1.28)
Sound environment (e.g., background noise, appropriate music)	4.00 (1.36)		4.09 (1.20)	*	4.28 (1.29)	*	3.94 (1.28)
Introduction of natural light	3.78 (1.36)		4.03 (1.23)	*	4.17 (1.32)	*	3.83 (1.29)
Appropriate lighting environment	4.10 (1.29)		4.25 (1.14)	*	4.45 (1.22)	*	4.02 (1.26)
Temperature setting (e.g., adjusted for the season)	4.42 (1.31)	*	4.52 (1.13)	*	4.73 (1.20)	*	4.23 (1.29)

Preferred location or seating (e.g., near a window or on a terrace)	4.00 (1.34)		4.21 (1.16)	*	4.32 (1.26)	*	3.99 (1.28)
Comfort of seating (e.g., chairs, height of tables)	4.35 (1.33)	*	4.38 (1.19)	*	4.62 (1.23)	*	4.17 (1.28)
Unique design or theme (e.g., scenery or culture)	3.50 (1.28)		3.76 (1.17)	*	3.77 (1.27)	*	3.61 (1.25)
Provision of privacy (e.g., private rooms or semi-private rooms)	4.22 (1.37)		4.31 (1.21)	*	4.40 (1.29)	*	4.11 (1.31)
Acceptance of all family members (e.g., with children or pets)	4.04 (1.50)		4.06 (1.31)		4.22 (1.42)	*	3.91 (1.38)
Digital environment (e.g., Wi-Fi, charging facilities)	3.74 (1.51)		3.91 (1.33)		3.89 (1.50)		3.84 (1.38)
Privacy of conversation	4.31 (1.37)		4.41 (1.19)	*	4.59 (1.27)	*	4.20 (1.33)
Modern ordering system (e.g., touch panels)	3.51 (1.31)		3.62 (1.21)		3.53 (1.31)		3.59 (1.26)
Visual information about ingredients and dishes (e.g., photos or descriptions)	4.02 (1.32)		4.05 (1.21)		4.19 (1.27)	*	3.93 (1.26)
Frequency of Snacking or Break Time (SA)		*		*		*	
More than twice a day	97 (11.5)		154 (9.4)		2384 (13.1)		423 (6.6)
Once a day	186 (22.0)		391 (23.8)		5217 (28.6)		1121 (17.5)
4-6 times a week	82 (9.7)		166 (10.1)		1636 (9.0)		617 (9.6)
2-3 times a week	138 (16.3)		256 (15.6)		2503 (13.7)		999 (15.6)
About once a week	59 (7.0)		137 (8.3)		1233 (6.8)		592 (9.2)
1-2 times a month	48 (5.7)		78 (4.8)		636 (3.5)		315 (4.9)
Rarely	235 (27.8)		459 (28.0)		4635 (25.4)		2357 (36.7)
Main Time for Snacking or Break Time							
Morning (after breakfast until before lunch)	73 (8.6)		149 (9.1)	*	2299 (12.6)	*	419 (6.5)
After lunch until 3 PM	176 (20.8)		352 (21.5)	*	4640 (25.4)	*	1060 (16.5)
3 PM until before dinner	309 (36.6)	*	591 (36.0)	*	7268 (39.8)	*	1939 (30.2)
After dinner	154 (18.2)		303 (18.5)		2706 (14.8)		1059 (16.5)
Late at night	52 (6.2)		51 (3.1)	*	525 (2.9)	*	335 (5.2)
No specific time	79 (9.3)		158 (9.6)		1323 (7.3)	*	558 (8.7)
Rarely	210 (24.9)	*	395 (24.1)	*	3961 (21.7)	*	2162 (33.7)

Selection of Foods Normally Not Chosen in Specific Situations or Contexts (SA)		*				*	
Often	54 (6.4)		51 (3.1)		882 (4.8)		205 (3.2)
Occasionally	229 (27.1)		403 (24.6)		4949 (27.1)		1381 (21.5)
Rarely	222 (26.3)		475 (28.9)		5212 (28.6)		1817 (28.3)
Almost never	340 (40.2)		712 (43.4)		7201 (39.5)		3021 (47.0)
**IV. Everyday meal experiences**							
Meals Usually Prepared by Oneself	543 (64.3)	*	928 (56.6)	*	11299 (61.9)	*	3269 (50.9)
Number of Days Eating Alone in the Past 7 Days							
Breakfast (SA)		*		*		*	
0 days	152 (18.0)		368 (22.4)		5972 (32.7)		1329 (20.7)
1-2 days	98 (11.6)		168 (10.2)		1822 (10.0)		800 (12.5)
3-4 days	64 (7.6)		151 (9.2)		1221 (6.7)		639 (9.9)
5-6 days	80 (9.5)		174 (10.6)		1425 (7.8)		634 (9.9)
Every day (7 days)	302 (35.7)		597 (36.4)		6371 (34.9)		1723 (26.8)
Usually do not eat breakfast	149 (17.6)		183 (11.2)		1433 (7.9)		1299 (20.2)
Lunch (SA)				*		*	
0 days	124 (14.7)		272 (16.6)		4515 (24.7)		983 (15.3)
1-2 days	135 (16.0)		284 (17.3)		2977 (16.3)		1033 (16.1)
3-4 days	108 (12.8)		229 (14.0)		2353 (12.9)		1016 (15.8)
5-6 days	154 (18.2)		300 (18.3)		2888 (15.8)		1037 (16.1)
Every day (7 days)	269 (31.8)		476 (29.0)		4798 (26.3)		1806 (28.1)
Usually do not eat lunch	55 (6.5)		80 (4.9)		713 (3.9)		549 (8.5)
Dinner (SA)				*		*	
0 days	329 (38.9)		692 (42.2)		9677 (53.0)		2218 (34.5)
1-2 days	108 (12.8)		235 (14.3)		2089 (11.5)		931 (14.5)
3-4 days	75 (8.9)		156 (9.5)		1228 (6.7)		693 (10.8)
5-6 days	102 (12.1)		144 (8.8)		1272 (7.0)		649 (10.1)
Every day (7 days)	214 (25.3)		448 (27.3)		7089 (38.9)		1328 (20.7)
Usually do not eat dinner	17 (2.0)		20 (1.2)		261 (1.4)		256 (4.0)
Frequently Experienced Conversation Situations							
Breakfast (SA)				*		*	
No conversation	531 (62.8)		884 (53.9)		8148 (44.7)		3816 (59.4)

Simple conversation: Basic greetings or essential communication only	184 (21.8)	447 (27.2)	5291 (29.0)	1500 (23.3)
Normal conversation: Everyday communication with friends or family	125 (14.8)	290 (17.7)	4527 (24.8)	1005 (15.6)
Deep/long conversation: Detailed discussion on specific themes or topics	5 (0.6)	20 (1.2)	278 (1.5)	103 (1.6)
Lunch (SA)				*
No conversation	434 (51.4)	726 (44.2)	6917 (37.9)	3185 (49.6)
Simple conversation: Basic greetings or essential communication only	185 (21.9)	448 (27.3)	4475 (24.5)	1570 (24.4)
Normal conversation: Everyday communication with friends or family	215 (25.4)	439 (26.8)	6500 (35.6)	1544 (24.0)
Deep/long conversation: Detailed discussion on specific themes or topics	11 (1.3)	22 (1.3)	359 (2.0)	232 (3.6)
Dinner (SA)			*	*
No conversation	262 (31.0)	416 (25.4)	4012 (22.0)	2254 (35.1)
Simple conversation: Basic greetings or essential communication only	161 (19.1)	405 (24.7)	3408 (18.7)	1402 (21.8)
Normal conversation: Everyday communication with friends or family	363 (43.0)	679 (41.4)	5397 (29.6)	3021 (47.0)
Deep/long conversation: Detailed discussion on specific themes or topics	59 (7.0)	91 (5.5)	1679 (9.2)	380 (5.9)
Satisfaction with Meals				
Breakfast (SA)			*	*
Not satisfied at all	38 (4.5)	42 (2.6)	440 (2.4)	392 (6.1)
Not very satisfied	86 (10.2)	130 (7.9)	1103 (6.0)	551 (8.6)
Neither satisfied nor dissatisfied	363 (43.0)	729 (44.4)	9145 (50.1)	2388 (37.2)
Somewhat satisfied	263 (31.1)	619 (37.7)	8200 (44.9)	1937 (30.2)
Very satisfied	95 (11.2)	171 (10.4)	3104 (17.0)	523 (8.1)
Lunch (SA)			*	*
Not satisfied at all	21 (2.5)	40 (2.4)	293 (1.6)	307 (4.8)

Not very satisfied	75 (8.9)	124 (7.6)	1074 (5.9)	579 (9.0)
Neither satisfied nor dissatisfied	355 (42.0)	628 (38.3)	5655 (31.0)	2830 (44.1)
Somewhat satisfied	306 (36.2)	683 (41.6)	8367 (45.9)	2127 (33.1)
Very satisfied	88 (10.4)	166 (10.1)	2855 (15.6)	581 (9.0)
Dinner (SA)		*	*	*
Not satisfied at all	13 (1.5)	28 (1.7)	255 (1.4)	259 (4.0)
Not very satisfied	56 (6.6)	78 (4.8)	818 (4.5)	490 (7.6)
Neither satisfied nor dissatisfied	264 (31.2)	445 (27.1)	3618 (19.8)	2307 (35.9)
Somewhat satisfied	379 (44.9)	802 (48.9)	8904 (48.8)	2549 (39.7)
Very satisfied	133 (15.7)	275 (16.8)	4744 (26.0)	840 (13.1)
Perceived Time Availability During Meals				
Breakfast (SA)			*	*
Not sufficient at all	81 (9.6)	95 (5.8)	1130 (6.2)	601 (9.4)
Not very sufficient	165 (19.5)	305 (18.6)	3118 (17.1)	1136 (17.7)
Neither sufficient nor insufficient	257 (30.4)	486 (29.6)	3821 (20.9)	2333 (36.3)
Somewhat sufficient	235 (27.8)	525 (32.0)	6107 (33.5)	1654 (25.7)
Very sufficient	107 (12.7)	230 (14.0)	4068 (22.3)	700 (10.9)
Lunch (SA)			*	*
Not sufficient at all	29 (3.4)	37 (2.3)	420 (2.3)	307 (4.8)
Not very sufficient	96 (11.4)	170 (10.4)	1606 (8.8)	725 (11.3)
Neither sufficient nor insufficient	279 (33.0)	502 (30.6)	4165 (22.8)	2344 (36.5)
Somewhat sufficient	294 (34.8)	651 (39.7)	7400 (40.6)	2213 (34.4)
Very sufficient	147 (17.4)	281 (17.1)	4653 (25.5)	835 (13.0)
Dinner (SA)		*	*	*
Not sufficient at all	14 (1.7)	28 (1.7)	296 (1.6)	213 (3.3)
Not very sufficient	56 (6.6)	91 (5.5)	723 (4.0)	469 (7.3)
Neither sufficient nor insufficient	202 (23.9)	346 (21.1)	2577 (14.1)	1835 (28.6)
Somewhat sufficient	359 (42.5)	741 (45.2)	7464 (40.9)	2558 (39.8)
Very sufficient	214 (25.3)	394 (24.0)	3717 (20.4)	1677 (26.1)
Frequency of Meal Types				
Homemade meals at home (SA)		*	*	*

Almost every day	433 (51.2)	757 (46.1)	12426 (68.1)	2212 (34.4)
3-4 times a week	160 (18.9)	358 (21.8)	2560 (14.0)	1342 (20.9)
1-2 times a week	94 (11.1)	203 (12.4)	1187 (6.5)	905 (14.1)
1-2 times a month	34 (4.0)	94 (5.7)	563 (3.1)	461 (7.2)
Once or twice every six months	24 (2.8)	51 (3.1)	535 (2.9)	267 (4.2)
Rarely/Never	100 (11.8)	179 (10.9)	1210 (6.6)	1208 (18.8)
Eating out (restaurants, fast food, etc.) (SA)		*	*	
Almost every day	16 (1.9)	12 (0.7)	216 (1.2)	107 (1.7)
3-4 times a week	41 (4.9)	39 (2.4)	554 (3.0)	277 (4.3)
1-2 times a week	157 (18.6)	278 (16.9)	2675 (14.7)	1052 (16.4)
1-2 times a month	284 (33.6)	573 (34.9)	6674 (36.6)	2067 (32.2)
Once or twice every six months	168 (19.9)	360 (21.9)	4106 (22.5)	1205 (18.8)
Rarely/Never	179 (21.2)	379 (23.1)	4019 (22.0)	1716 (26.7)
Takeout (bento, onigiri, etc.) (SA)		*	*	
Almost every day	25 (3.0)	28 (1.7)	396 (2.2)	182 (2.8)
3-4 times a week	63 (7.5)	95 (5.8)	843 (4.6)	444 (6.9)
1-2 times a week	139 (16.4)	250 (15.2)	2452 (13.4)	1198 (18.6)
1-2 times a month	214 (25.3)	474 (28.9)	4481 (24.6)	1525 (23.7)
Once or twice every six months	143 (16.9)	266 (16.2)	3426 (18.8)	961 (15.0)
Rarely/Never	261 (30.9)	528 (32.2)	6646 (36.4)	2114 (32.9)
Delivery services (SA)		*	*	
Almost every day	6 (0.7)	4 (0.2)	176 (1.0)	53 (0.8)
3-4 times a week	9 (1.1)	8 (0.5)	245 (1.3)	135 (2.1)
1-2 times a week	28 (3.3)	57 (3.5)	457 (2.5)	345 (5.4)
1-2 times a month	55 (6.5)	100 (6.1)	880 (4.8)	516 (8.0)
Once or twice every six months	85 (10.1)	162 (9.9)	1637 (9.0)	672 (10.5)
Rarely/Never	662 (78.3)	1304 (79.5)	14902 (81.7)	4651 (72.4)
Eat-in at convenience stores or shopping malls (SA)		*	*	
Almost every day	11 (1.3)	12 (0.7)	188 (1.0)	85 (1.3)
3-4 times a week	22 (2.6)	30 (1.8)	394 (2.2)	262 (4.1)
1-2 times a week	61 (7.2)	108 (6.6)	844 (4.6)	535 (8.3)

1-2 times a month	143 (16.9)	267 (16.3)	2528 (13.9)	983 (15.3)
Once or twice every six months	158 (18.7)	335 (20.4)	3621 (19.8)	1183 (18.4)
Rarely/Never	450 (53.3)	837 (51.0)	7486 (41.0)	3799 (59.1)
Prepared foods from supermarkets (SA)		*	*	
Almost every day	32 (3.8)	44 (2.7)	556 (3.0)	162 (2.5)
3-4 times a week	97 (11.5)	172 (10.5)	1682 (9.2)	637 (9.9)
1-2 times a week	269 (31.8)	546 (33.3)	5313 (29.1)	1890 (29.4)
1-2 times a month	210 (24.9)	457 (27.8)	5197 (28.5)	1652 (25.7)
Once or twice every six months	100 (11.8)	164 (10.0)	2140 (11.7)	737 (11.5)
Rarely/Never	137 (16.2)	258 (15.7)	3356 (18.4)	1346 (21.0)
Light meals at cafes or tea shops (SA)		*	*	
Almost every day	6 (0.7)	1 (0.1)	153 (0.8)	69 (1.1)
3-4 times a week	15 (1.8)	25 (1.5)	292 (1.6)	162 (2.5)
1-2 times a week	48 (5.7)	106 (6.5)	1110 (6.1)	549 (8.5)
1-2 times a month	128 (15.1)	276 (16.8)	3151 (17.3)	1088 (16.9)
Once or twice every six months	197 (23.3)	375 (22.9)	4061 (22.3)	1210 (18.8)
Rarely/Never	451 (53.4)	852 (51.9)	9476 (51.9)	3343 (52.0)
School or company cafeterias/canteens (SA)	*	*	*	
Almost every day	23 (2.7)	38 (2.3)	480 (2.6)	199 (3.1)
3-4 times a week	42 (5.0)	106 (6.5)	1258 (6.9)	391 (6.1)
1-2 times a week	36 (4.3)	53 (3.2)	646 (3.5)	403 (6.3)
1-2 times a month	32 (3.8)	67 (4.1)	505 (2.8)	364 (5.7)
Once or twice every six months	21 (2.5)	43 (2.6)	540 (3.0)	339 (5.3)
Rarely/Never	691 (81.8)	1358 (82.8)	15360 (84.2)	4795 (74.6)
Instant food (SA)		*	*	
Almost every day	24 (2.8)	12 (0.7)	364 (2.0)	160 (2.5)
3-4 times a week	55 (6.5)	87 (5.3)	972 (5.3)	469 (7.3)
1-2 times a week	226 (26.7)	454 (27.7)	4315 (23.7)	1712 (26.7)
1-2 times a month	295 (34.9)	601 (36.6)	6178 (33.9)	1934 (30.1)
Once or twice every six months	104 (12.3)	223 (13.6)	2828 (15.5)	813 (12.7)

Rarely/Never	141 (16.7)	264 (16.1)	3587 (19.7)	1336 (20.8)
Frozen food (SA)		*	*	
Almost every day	30 (3.6)	28 (1.7)	616 (3.4)	150 (2.3)
3-4 times a week	81 (9.6)	128 (7.8)	1834 (10.1)	571 (8.9)
1-2 times a week	223 (26.4)	513 (31.3)	5219 (28.6)	1710 (26.6)
1-2 times a month	238 (28.2)	486 (29.6)	5108 (28.0)	1688 (26.3)
Once or twice every six months	99 (11.7)	198 (12.1)	2121 (11.6)	809 (12.6)
Rarely/Never	174 (20.6)	288 (17.6)	3346 (18.3)	1496 (23.3)
Frequency of Methods for Purchasing Ingredients and Groceries				
Supermarket (SA)	*	*	*	
Almost every day	96 (11.4)	146 (8.9)	2479 (13.6)	472 (7.3)
3-4 times a week	214 (25.3)	468 (28.5)	5363 (29.4)	1394 (21.7)
1-2 times a week	387 (45.8)	767 (46.7)	8175 (44.8)	2758 (42.9)
1-2 times a month	78 (9.2)	140 (8.5)	1259 (6.9)	779 (12.1)
Once or twice every six months	17 (2.0)	33 (2.0)	322 (1.8)	265 (4.1)
Rarely/Never	53 (6.3)	87 (5.3)	646 (3.5)	756 (11.8)
Convenience store (SA)			*	
Almost every day	26 (3.1)	58 (3.5)	396 (2.2)	251 (3.9)
3-4 times a week	69 (8.2)	93 (5.7)	899 (4.9)	491 (7.6)
1-2 times a week	155 (18.3)	293 (17.9)	2689 (14.7)	1247 (19.4)
1-2 times a month	202 (23.9)	385 (23.5)	4161 (22.8)	1449 (22.6)
Once or twice every six months	137 (16.2)	264 (16.1)	3242 (17.8)	925 (14.4)
Rarely/Never	256 (30.3)	577 (35.2)	6879 (37.7)	2140 (33.3)
Local market or direct sales outlet (SA)		*	*	
Almost every day	6 (0.7)	7 (0.4)	154 (0.8)	72 (1.1)
3-4 times a week	11 (1.3)	19 (1.2)	480 (2.6)	172 (2.7)
1-2 times a week	54 (6.4)	109 (6.6)	1788 (9.8)	533 (8.3)
1-2 times a month	87 (10.3)	234 (14.3)	2948 (16.2)	794 (12.4)
Once or twice every six months	152 (18.0)	295 (18.0)	3525 (19.3)	1008 (15.7)
Rarely/Never	535 (63.3)	980 (59.7)	9327 (51.1)	3864 (60.1)

Specialty stores (butcher, fishmonger, greengrocer, etc.) (SA)			*	
Almost every day	9 (1.1)	7 (0.4)	198 (1.1)	50 (0.8)
3-4 times a week	18 (2.1)	37 (2.3)	597 (3.3)	173 (2.7)
1-2 times a week	48 (5.7)	134 (8.2)	2020 (11.1)	581 (9.0)
1-2 times a month	94 (11.1)	243 (14.8)	2741 (15.0)	848 (13.2)
Once or twice every six months	110 (13.0)	249 (15.2)	2725 (14.9)	910 (14.2)
Rarely/Never	566 (67.0)	971 (59.2)	9963 (54.6)	3862 (60.1)
Online shop (SA)	*	*	*	
Almost every day	5 (0.6)	4 (0.2)	127 (0.7)	46 (0.7)
3-4 times a week	6 (0.7)	8 (0.5)	164 (0.9)	107 (1.7)
1-2 times a week	32 (3.8)	56 (3.4)	819 (4.5)	385 (6.0)
1-2 times a month	64 (7.6)	181 (11.0)	2163 (11.9)	728 (11.3)
Once or twice every six months	118 (14.0)	254 (15.5)	2898 (15.9)	841 (13.1)
Rarely/Never	620 (73.4)	1135 (69.2)	12023 (65.9)	4288 (66.7)
Meal kits (SA)	*	*	*	
Almost every day	5 (0.6)	3 (0.2)	112 (0.6)	40 (0.6)
3-4 times a week	9 (1.1)	10 (0.6)	215 (1.2)	113 (1.8)
1-2 times a week	16 (1.9)	19 (1.2)	217 (1.2)	201 (3.1)
1-2 times a month	27 (3.2)	72 (4.4)	706 (3.9)	382 (5.9)
Once or twice every six months	35 (4.1)	107 (6.5)	1108 (6.1)	439 (6.8)
Rarely/Never	753 (89.1)	1408 (85.8)	15731 (86.2)	5106 (79.5)
Direct purchase from farmers or producers (SA)	*	*	*	
Almost every day	4 (0.5)	3 (0.2)	110 (0.6)	42 (0.7)
3-4 times a week	7 (0.8)	12 (0.7)	316 (1.7)	138 (2.1)
1-2 times a week	43 (5.1)	61 (3.7)	1058 (5.8)	366 (5.7)
1-2 times a month	46 (5.4)	123 (7.5)	1848 (10.1)	538 (8.4)
Once or twice every six months	72 (8.5)	200 (12.2)	2364 (13.0)	686 (10.7)
Rarely/Never	673 (79.6)	1243 (75.7)	12527 (68.7)	4650 (72.4)
Organic or pesticide-free specialty stores or online shops (SA)	*	*	*	

Almost every day	5 (0.6)	3 (0.2)	120 (0.7)	47 (0.7)
3-4 times a week	9 (1.1)	12 (0.7)	253 (1.4)	144 (2.2)
1-2 times a week	17 (2.0)	37 (2.3)	576 (3.2)	319 (5.0)
1-2 times a month	17 (2.0)	75 (4.6)	804 (4.4)	371 (5.8)
Once or twice every six months	35 (4.1)	94 (5.7)	889 (4.9)	424 (6.6)
Rarely/Never	762 (90.2)	1411 (86.0)	15399 (84.4)	5120 (79.7)
Large discount stores (SA)		*	*	*
Almost every day	4 (0.5)	1 (0.1)	95 (0.5)	41 (0.6)
3-4 times a week	11 (1.3)	22 (1.3)	285 (1.6)	134 (2.1)
1-2 times a week	26 (3.1)	84 (5.1)	795 (4.4)	406 (6.3)
1-2 times a month	81 (9.6)	143 (8.7)	1678 (9.2)	663 (10.3)
Once or twice every six months	108 (12.8)	249 (15.2)	2633 (14.4)	945 (14.7)
Rarely/Never	615 (72.8)	1140 (69.5)	12709 (69.7)	4218 (65.7)
Most Common Mode of Transportation for Grocery Shopping (SA)				*
Walking	217 (25.7)	420 (25.6)	4736 (26.0)	1873 (29.2)
Bicycle	138 (16.3)	258 (15.7)	3042 (16.7)	1041 (16.2)
Self-driven car or motorcycle	411 (48.6)	812 (49.5)	8428 (46.2)	2850 (44.4)
Car or motorcycle driven by someone else	65 (7.7)	125 (7.6)	1635 (9.0)	480 (7.5)
Bus, taxi, train	12 (1.4)	22 (1.3)	331 (1.8)	159 (2.5)
Other	2 (0.2)	4 (0.2)	72 (0.4)	21 (0.3)
Frequency of Participation in Social Dining or Food-centered Events				
Office or school lunch gatherings (SA)			*	*
Almost every day	3 (0.4)	3 (0.2)	122 (0.7)	42 (0.7)
3-4 times a week	9 (1.1)	17 (1.0)	215 (1.2)	171 (2.7)
1-2 times a week	20 (2.4)	18 (1.1)	295 (1.6)	243 (3.8)
1-2 times a month	25 (3.0)	50 (3.0)	539 (3.0)	311 (4.8)
Once or twice every six months	44 (5.2)	96 (5.9)	1040 (5.7)	419 (6.5)
Rarely/Never	744 (88.0)	1388 (84.6)	15207 (83.4)	5233 (81.5)

Dining out with friends or colleagues (SA)			*	*
Almost every day	1 (0.1)	1 (0.1)	102 (0.6)	41 (0.6)
3-4 times a week	11 (1.3)	26 (1.6)	315 (1.7)	123 (1.9)
1-2 times a week	39 (4.6)	66 (4.0)	936 (5.1)	448 (7.0)
1-2 times a month	160 (18.9)	298 (18.2)	3677 (20.2)	1117 (17.4)
Once or twice every six months	234 (27.7)	490 (29.9)	5704 (31.3)	1528 (23.8)
Rarely/Never	400 (47.3)	773 (47.1)	7589 (41.6)	3159 (49.2)
Special occasion meals with family or friends (birthdays, weddings, etc.) (SA)		*	*	*
Almost every day	1 (0.1)	3 (0.2)	114 (0.6)	42 (0.7)
3-4 times a week	5 (0.6)	8 (0.5)	148 (0.8)	110 (1.7)
1-2 times a week	15 (1.8)	21 (1.3)	315 (1.7)	226 (3.5)
1-2 times a month	58 (6.9)	104 (6.3)	1448 (7.9)	506 (7.9)
Once or twice every six months	340 (40.2)	666 (40.6)	8443 (46.3)	2018 (31.4)
Rarely/Never	426 (50.4)	839 (51.1)	7776 (42.6)	3522 (54.8)
Outdoor dining like barbecues or picnics (SA)			*	*
Almost every day	4 (0.5)	1 (0.1)	94 (0.5)	38 (0.6)
3-4 times a week	8 (0.9)	6 (0.4)	153 (0.8)	112 (1.7)
1-2 times a week	13 (1.5)	16 (1.0)	269 (1.5)	209 (3.3)
1-2 times a month	15 (1.8)	26 (1.6)	464 (2.5)	243 (3.8)
Once or twice every six months	121 (14.3)	259 (15.8)	3097 (17.0)	923 (14.4)
Rarely/Never	684 (80.9)	1324 (80.7)	14188 (77.8)	4891 (76.1)
Online meal gatherings (SA)		*	*	*
Almost every day	4 (0.5)	2 (0.1)	131 (0.7)	46 (0.7)
3-4 times a week	6 (0.7)	8 (0.5)	146 (0.8)	106 (1.7)
1-2 times a week	14 (1.7)	28 (1.7)	340 (1.9)	254 (4.0)
1-2 times a month	11 (1.3)	28 (1.7)	352 (1.9)	125 (1.9)
Once or twice every six months	24 (2.8)	50 (3.0)	298 (1.6)	296 (4.6)
Rarely/Never	786 (93.0)	1533 (93.4)	16847 (92.3)	5575 (86.8)
Cooking classes or food workshops (SA)		*	*	*

Almost every day	2 (0.2)	2 (0.1)	100 (0.5)	37 (0.6)
3-4 times a week	6 (0.7)	8 (0.5)	150 (0.8)	100 (1.6)
1-2 times a week	8 (0.9)	16 (1.0)	241 (1.3)	198 (3.1)
1-2 times a month	19 (2.2)	31 (1.9)	390 (2.1)	228 (3.5)
Once or twice every six months	22 (2.6)	51 (3.1)	561 (3.1)	298 (4.6)
Rarely/Never	788 (93.3)	1533 (93.4)	16806 (92.1)	5557 (86.5)
Casual meetings or study groups in cafes or tea shops (SA)	*	*	*	
Almost every day	4 (0.5)	0 (0.0)	110 (0.6)	44 (0.7)
3-4 times a week	4 (0.5)	10 (0.6)	161 (0.9)	100 (1.6)
1-2 times a week	14 (1.7)	24 (1.5)	229 (1.3)	201 (3.1)
1-2 times a month	23 (2.7)	61 (3.7)	856 (4.7)	318 (5.0)
Once or twice every six months	56 (6.6)	154 (9.4)	1570 (8.6)	475 (7.4)
Rarely/Never	744 (88.0)	1464 (89.2)	16033 (87.9)	5296 (82.4)
Catered meals at events or parties (SA)	*	*	*	
Almost every day	4 (0.5)	3 (0.2)	144 (0.8)	58 (0.9)
3-4 times a week	3 (0.4)	10 (0.6)	166 (0.9)	94 (1.5)
1-2 times a week	16 (1.9)	51 (3.1)	538 (2.9)	322 (5.0)
1-2 times a month	13 (1.5)	28 (1.7)	413 (2.3)	253 (3.9)
Once or twice every six months	52 (6.2)	116 (7.1)	1259 (6.9)	471 (7.3)
Rarely/Never	757 (89.6)	1467 (89.4)	16095 (88.2)	5367 (83.5)
After-work events with meals or drinks (SA)		*	*	
Almost every day	3 (0.4)	3 (0.2)	99 (0.5)	38 (0.6)
3-4 times a week	10 (1.2)	11 (0.7)	166 (0.9)	114 (1.8)
1-2 times a week	12 (1.4)	18 (1.1)	377 (2.1)	275 (4.3)
1-2 times a month	53 (6.3)	102 (6.2)	1163 (6.4)	456 (7.1)
Once or twice every six months	129 (15.3)	261 (15.9)	2806 (15.4)	851 (13.2)
Rarely/Never	638 (75.5)	1248 (76.1)	13642 (74.8)	4686 (72.9)
Community or local food events (SA)	*	*	*	
Almost every day	3 (0.4)	1 (0.1)	90 (0.5)	42 (0.7)
3-4 times a week	6 (0.7)	12 (0.7)	221 (1.2)	135 (2.1)

1-2 times a week	13 (1.5)		24 (1.5)		330 (1.8)		146 (2.3)
1-2 times a month	16 (1.9)		37 (2.3)		478 (2.6)		252 (3.9)
Once or twice every six months	57 (6.7)		137 (8.3)		1630 (8.9)		505 (7.9)
Rarely/Never	750 (88.8)		1440 (87.8)		15618 (85.6)		5320 (82.8)
Use of Technology or Apps Related to Food and Ingredients							
Recipe suggestion apps (e.g., Cookpad, Delish Kitchen, etc.)	201 (23.8)	*	277 (16.9)	*	4381 (24.0)	*	787 (12.3)
Calorie and nutrition management apps (e.g., MyFitnessPal, AskKen, etc.)	30 (3.6)		31 (1.9)		611 (3.3)		193 (3.0)
Inventory and expiration date management apps for ingredients and groceries	7 (0.8)		28 (1.7)		393 (2.2)		142 (2.2)
Food waste prevention apps (e.g., notifications about ingredients and groceries nearing expiration, discounted food notifications)	12 (1.4)		23 (1.4)		437 (2.4)		154 (2.4)
Delivery service apps for ingredients (e.g., Oisix, Rakuten Mart, etc.)	12 (1.4)		34 (2.1)		541 (3.0)		151 (2.4)
Restaurant reservation and review apps (e.g., Tabelog, Retty, etc.)	144 (17.0)		284 (17.3)	*	3713 (20.4)	*	850 (13.2)
Apps providing information on the origin and sustainability of ingredients (e.g., CodeCheck, etc.)	9 (1.1)		28 (1.7)		344 (1.9)		105 (1.6)
Diet support apps for avoiding allergies or specific ingredients	4 (0.5)		21 (1.3)		270 (1.5)		108 (1.7)
Control and management apps for smart kitchen devices (e.g., IoT refrigerators, smart ovens, etc.)	8 (0.9)		24 (1.5)		317 (1.7)		134 (2.1)
AR (Augmented Reality) cooking support apps	7 (0.8)		12 (0.7)		172 (0.9)		74 (1.2)
Apps for purchasing regional specialties (e.g., selecting return gifts for Furusato	24 (2.8)		67 (4.1)		856 (4.7)	*	205 (3.2)

Nozei, specialty product market apps, etc.)							
Apps for learning new recipes or improving cooking skills (e.g., Udemy, YouTube channels, etc.)	66 (7.8)		90 (5.5)		1774 (9.7)	*	359 (5.6)
Map apps (e.g., Google Maps, etc.)	185 (21.9)	*	305 (18.6)		4205 (23.0)	*	1008 (15.7)
None apply	437 (51.7)	*	966 (58.9)	*	9252 (50.7)	*	4101 (63.8)
Other	0 (0.0)		0 (0.0)		21 (0.1)		7 (0.1)
Changes in Your Food and Ingredients-Related Habits After the COVID-19 Pandemic							
Emphasizing safety and hygiene management in choosing ingredients and groceries	54 (6.4)		162 (9.9)		2494 (13.7)	*	495 (7.7)
Decrease in frequency of dining out and increase in frequency of cooking at home	140 (16.6)	*	260 (15.8)	*	4031 (22.1)	*	714 (11.1)
Increase in use of online shopping and delivery services for ingredients and groceries	42 (5.0)		62 (3.8)		997 (5.5)		286 (4.5)
Stockpiling long-lasting food items (canned goods, frozen foods, etc.)	105 (12.4)		200 (12.2)		3037 (16.6)	*	601 (9.4)
Interest in home gardening or urban agriculture and starting to practice it	22 (2.6)		58 (3.5)		1086 (6.0)	*	195 (3.0)
Increased awareness of supporting and purchasing locally produced ingredients and groceries	35 (4.1)		108 (6.6)		1843 (10.1)	*	318 (5.0)
Concern about the hygiene management and crowding of restaurants when dining out	106 (12.5)		195 (11.9)		3145 (17.2)	*	623 (9.7)
Increased interest in ingredients and groceries or supplements that boost immunity	57 (6.7)		153 (9.3)		2540 (13.9)	*	500 (7.8)
Gathering and learning information about the	12 (1.4)		47 (2.9)		609 (3.3)		170 (2.6)

supply chain and distribution of food and groceries							
Increased participation and interest in online cooking classes and meal gatherings	7 (0.8)	17 (1.0)		283 (1.6)		113 (1.8)
Efforts to reduce food waste and increase awareness of reuse	95 (11.2)	197 (12.0)	*	2986 (16.4)	*	555 (8.6)
Preference for restaurants and cafes that introduce contactless payment and automated serving	27 (3.2)	46 (2.8)		651 (3.6)		232 (3.6)
Interest in recipes that shorten cooking time and simple cooking foods	81 (9.6)	142 (8.7)		1857 (10.2)	*	426 (6.6)
Spending more time and effort on cooking, trying new recipes, and authentic dishes	23 (2.7)	55 (3.4)		1055 (5.8)	*	237 (3.7)
None apply	471 (55.7)	879 (53.6)	*	8327 (45.6)	*	3876 (60.3)
Other	2 (0.2)	0 (0.0)		19 (0.1)		6 (0.1)

SA refers to single-answer questions. The asterisk (*) indicates that the test result is statistically significant when comparing with reference Cluster 4; the Bonferroni correction was applied for multiple testing as needed.

Supplementary Table S3: Results of the survey for Outcome 2 by clusters: number (%)

	Cluster A		Cluster B		Cluster C		Cluster D
	n=1483, 5.46%		n=2573, 9.48%		n=17401, 64.08%		n=5697, 20.98%
Variables							
**I. Sociodemographic characteristics**							
Age, mean (standard deviation)	46.30 (16.54)	*	45.13 (16.09)	*	56.73 (15.94)	*	49.76 (16.11)
Gender (SA)		*				*	
Female	695 (46.9)		1111 (43.2)		8830 (50.7)		2367 (41.5)
Male	766 (51.7)		1437 (55.8)		8493 (48.8)		3301 (57.9)
Other	22 (1.5)		25 (1.0)		78 (0.4)		29 (0.5)
Body-mass index (BMI), mean (standard deviation)	21.68 (3.39)		21.61 (3.18)		21.99 (3.14)	*	21.75 (3.21)
Residence (SA)						*	
Hokkaido	54 (3.6)		100 (3.9)		686 (3.9)		239 (4.2)
Tohoku							
Aomori	20 (1.3)		44 (1.7)		173 (1.0)		61 (1.1)
Iwate	13 (0.9)		16 (0.6)		153 (0.9)		56 (1.0)
Miyagi	24 (1.6)		59 (2.3)		417 (2.4)		98 (1.7)
Akita	11 (0.7)		23 (0.9)		154 (0.9)		49 (0.9)
Yamagata	21 (1.4)		32 (1.2)		150 (0.9)		40 (0.7)
Fukushima	15 (1.0)		38 (1.5)		210 (1.2)		42 (0.7)
Kanto							
Ibaraki	37 (2.5)		39 (1.5)		269 (1.5)		105 (1.8)
Tochigi	11 (0.7)		40 (1.6)		159 (0.9)		62 (1.1)
Gunma	16 (1.1)		41 (1.6)		184 (1.1)		68 (1.2)
Saitama	99 (6.7)		150 (5.8)		915 (5.3)		331 (5.8)
Chiba	85 (5.7)		135 (5.2)		847 (4.9)		268 (4.7)
Tokyo	217 (14.6)		324 (12.6)		2219 (12.8)		758 (13.3)
Kanagawa	111 (7.5)		186 (7.2)		1370 (7.9)		437 (7.7)
Chubu							
Niigata	19 (1.3)		32 (1.2)		261 (1.5)		96 (1.7)
Toyama	8 (0.5)		19 (0.7)		140 (0.8)		39 (0.7)
Ishikawa	14 (0.9)		23 (0.9)		155 (0.9)		53 (0.9)
Fukui	5 (0.3)		6 (0.2)		107 (0.6)		23 (0.4)

Yamanashi	1 (0.1)	17 (0.7)	91 (0.5)	43 (0.8)
Nagano	21 (1.4)	45 (1.7)	293 (1.7)	68 (1.2)
Gifu	20 (1.3)	34 (1.3)	298 (1.7)	105 (1.8)
Shizuoka	32 (2.2)	48 (1.9)	464 (2.7)	162 (2.8)
Aichi	107 (7.2)	180 (7.0)	1276 (7.3)	412 (7.2)
Kinki				
Mie	13 (0.9)	25 (1.0)	209 (1.2)	57 (1.0)
Shiga	15 (1.0)	28 (1.1)	155 (0.9)	57 (1.0)
Kyoto	28 (1.9)	54 (2.1)	342 (2.0)	107 (1.9)
Osaka	96 (6.5)	178 (6.9)	1166 (6.7)	469 (8.2)
Hyogo	53 (3.6)	111 (4.3)	763 (4.4)	247 (4.3)
Nara	8 (0.5)	25 (1.0)	159 (0.9)	64 (1.1)
Wakayama	15 (1.0)	19 (0.7)	95 (0.5)	38 (0.7)
Chugoku				
Tottori	7 (0.5)	9 (0.3)	83 (0.5)	20 (0.4)
Shimane	4 (0.3)	18 (0.7)	73 (0.4)	30 (0.5)
Okayama	24 (1.6)	45 (1.7)	277 (1.6)	90 (1.6)
Hiroshima	37 (2.5)	72 (2.8)	438 (2.5)	134 (2.4)
Yamaguchi	11 (0.7)	26 (1.0)	176 (1.0)	57 (1.0)
Shikoku				
Tokushima	12 (0.8)	13 (0.5)	105 (0.6)	33 (0.6)
Kagawa	15 (1.0)	17 (0.7)	180 (1.0)	26 (0.5)
Ehime	14 (0.9)	26 (1.0)	178 (1.0)	60 (1.1)
Kochi	3 (0.2)	10 (0.4)	76 (0.4)	29 (0.5)
Kyushu				
Fukuoka	72 (4.9)	126 (4.9)	917 (5.3)	255 (4.5)
Saga	12 (0.8)	16 (0.6)	94 (0.5)	32 (0.6)
Nagasaki	19 (1.3)	19 (0.7)	188 (1.1)	52 (0.9)
Kumamoto	15 (1.0)	31 (1.2)	185 (1.1)	55 (1.0)
Oita	10 (0.7)	18 (0.7)	125 (0.7)	52 (0.9)
Miyazaki	6 (0.4)	15 (0.6)	112 (0.6)	32 (0.6)
Kagoshima	17 (1.1)	21 (0.8)	182 (1.0)	42 (0.7)
Okinawa	16 (1.1)	20 (0.8)	132 (0.8)	44 (0.8)
Educational Background (SA)	*		*	
Junior high school graduate	89 (6.0)	114 (4.4)	258 (1.5)	140 (2.5)

High school / technical college graduate or enrolled	475 (32.0)	827 (32.1)	5714 (32.8)	1965 (34.5)
Junior college / vocational school graduate or enrolled	259 (17.5)	433 (16.8)	3419 (19.6)	946 (16.6)
University graduate or enrolled	572 (38.6)	1088 (42.3)	7195 (41.3)	2351 (41.3)
Graduate school completed or enrolled	86 (5.8)	133 (5.2)	726 (4.2)	234 (4.1)
Occupation (SA)			*	*
Managerial occupation	101 (6.8)	156 (6.1)	1070 (6.1)	345 (6.1)
Professional or technical occupation	154 (10.4)	320 (12.4)	1880 (10.8)	666 (11.7)
Clerical worker	193 (13.0)	376 (14.6)	2148 (12.3)	787 (13.8)
Sales worker	92 (6.2)	159 (6.2)	749 (4.3)	298 (5.2)
Service worker	171 (11.5)	316 (12.3)	1650 (9.5)	661 (11.6)
Security worker	16 (1.1)	31 (1.2)	118 (0.7)	58 (1.0)
Agriculture, forestry, and fisheries worker	11 (0.7)	25 (1.0)	102 (0.6)	43 (0.8)
Production process worker	83 (5.6)	163 (6.3)	609 (3.5)	313 (5.5)
Transport and machinery operation worker	21 (1.4)	43 (1.7)	164 (0.9)	79 (1.4)
Construction and mining worker	18 (1.2)	59 (2.3)	157 (0.9)	93 (1.6)
Material moving, cleaning, packing, etc. worker	40 (2.7)	73 (2.8)	315 (1.8)	145 (2.5)
Student	44 (3.0)	83 (3.2)	196 (1.1)	112 (2.0)
Full-time homemaker	185 (12.5)	280 (10.9)	3419 (19.6)	719 (12.6)
Other (including unemployed, retired)	354 (23.9)	489 (19.0)	4824 (27.7)	1378 (24.2)
Annual Income (SA)		*		*
Less than 2 million yen / about Less than $15,000	357 (24.1)	460 (17.9)	2654 (15.3)	1121 (19.7)
2 to under 4 million yen / about $15,400-$31,000	379 (25.6)	671 (26.1)	5329 (30.6)	1552 (27.2)
4 to under 6 million yen / about $30,800-$46,000	276 (18.6)	583 (22.7)	3933 (22.6)	1249 (21.9)
6 to under 8 million yen / about $46,200-$62,000	195 (13.1)	399 (15.5)	2462 (14.1)	795 (14.0)
8 to under 10 million yen / about $61,500-$77,000	98 (6.6)	234 (9.1)	1423 (8.2)	490 (8.6)

10 to under 20 million yen / about $76,900-$154,000	122 (8.2)	173 (6.7)	1364 (7.8)	399 (7.0)
Over 20 million yen / Over $154,000	56 (3.8)	53 (2.1)	236 (1.4)	91 (1.6)
Marital Status (SA)	*	*	*	
Married (including common-law marriage)	675 (45.5)	1318 (51.2)	11333 (65.1)	3044 (53.4)
Single (no partner)	523 (35.3)	808 (31.4)	3364 (19.3)	1767 (31.0)
Single (with a partner)	83 (5.6)	367 (14.3)	998 (5.7)	414 (7.3)
Widowed	43 (2.9)	52 (2.0)	726 (4.2)	157 (2.8)
Divorced	108 (7.3)	165 (6.4)	1151 (6.6)	372 (6.5)
Smoking (SA)		*	*	
Smokes daily	292 (19.7)	559 (21.7)	2860 (16.4)	1208 (21.2)
Smokes occasionally	22 (1.5)	95 (3.7)	249 (1.4)	88 (1.5)
Used to smoke but has not smoked for over a month	189 (12.7)	356 (13.8)	3446 (19.8)	876 (15.4)
Does not smoke	980 (66.1)	1563 (60.7)	10846 (62.3)	3525 (61.9)
Drinking (SA)	*		*	
Daily	250 (16.9)	401 (15.6)	2899 (16.7)	945 (16.6)
5-6 days per week	66 (4.5)	258 (10.0)	607 (3.5)	230 (4.0)
3-4 days per week	71 (4.8)	167 (6.5)	1201 (6.9)	305 (5.4)
1-2 days per week	125 (8.4)	284 (11.0)	2096 (12.0)	570 (10.0)
1-3 days per month	118 (8.0)	245 (9.5)	1670 (9.6)	541 (9.5)
Rarely drinks	187 (12.6)	382 (14.8)	2899 (16.7)	828 (14.5)
Stopped drinking	58 (3.9)	77 (3.0)	608 (3.5)	177 (3.1)
Does not drink (cannot drink)	619 (41.7)	851 (33.1)	4760 (27.4)	2023 (35.5)
Health Condition (SA)	*	*	*	
Good	335 (22.6)	432 (16.8)	2523 (14.5)	855 (15.0)
Fairly good	279 (18.8)	735 (28.6)	5109 (29.4)	1331 (23.4)
Average	505 (34.1)	932 (36.2)	7002 (40.2)	2632 (46.2)
Not very good	199 (13.4)	331 (12.9)	2377 (13.7)	686 (12.0)
Poor	165 (11.1)	143 (5.6)	390 (2.2)	193 (3.4)
Frequency of Device Use				
Wearable devices (SA)	*	*	*	
Almost every day	135 (9.1)	176 (6.8)	1251 (7.2)	317 (5.6)
2-5 days per week	35 (2.4)	151 (5.9)	149 (0.9)	56 (1.0)

About once a week or less	28 (1.9)	100 (3.9)	272 (1.6)	74 (1.3)
Do not use	1302 (87.8)	2193 (85.2)	15603 (89.7)	5228 (91.8)
IOT appliances (SA)		*	*	*
Almost every day	90 (6.1)	96 (3.7)	526 (3.0)	121 (2.1)
2-5 days per week	23 (1.6)	92 (3.6)	232 (1.3)	55 (1.0)
About once a week or less	20 (1.3)	226 (8.8)	121 (0.7)	132 (2.3)
Do not use	1346 (90.8)	2264 (88.0)	16360 (94.0)	5436 (95.4)
Frequency of Social Media Use				
Facebook (SA)			*	*
Almost every day	163 (11.0)	270 (10.5)	1547 (8.9)	471 (8.3)
2-5 days per week	53 (3.6)	161 (6.3)	943 (5.4)	217 (3.8)
About once a week or less	151 (10.2)	259 (10.1)	1962 (11.3)	533 (9.4)
Do not use	1116 (75.3)	1883 (73.2)	12949 (74.4)	4476 (78.6)
X/Twitter (SA)			*	*
Almost every day	456 (30.7)	757 (29.4)	3556 (20.4)	1509 (26.5)
2-5 days per week	98 (6.6)	236 (9.2)	1324 (7.6)	391 (6.9)
About once a week or less	113 (7.6)	283 (11.0)	1592 (9.1)	456 (8.0)
Do not use	816 (55.0)	1297 (50.4)	10929 (62.8)	3341 (58.6)
LINE (SA)			*	*
Almost every day	799 (53.9)	1452 (56.4)	9442 (54.3)	3001 (52.7)
2-5 days per week	162 (10.9)	398 (15.5)	2777 (16.0)	772 (13.6)
About once a week or less	135 (9.1)	277 (10.8)	1857 (10.7)	578 (10.1)
Do not use	387 (26.1)	446 (17.3)	3325 (19.1)	1346 (23.6)
Instagram (SA)		*	*	*
Almost every day	424 (28.6)	774 (30.1)	3592 (20.6)	1301 (22.8)
2-5 days per week	90 (6.1)	217 (8.4)	1224 (7.0)	312 (5.5)
About once a week or less	111 (7.5)	223 (8.7)	1575 (9.1)	433 (7.6)
Do not use	858 (57.9)	1359 (52.8)	11010 (63.3)	3651 (64.1)
Youtube (SA)		*	*	*
Almost every day	687 (46.3)	1144 (44.5)	6116 (35.1)	2181 (38.3)
2-5 days per week	184 (12.4)	426 (16.6)	3127 (18.0)	911 (16.0)
About once a week or less	203 (13.7)	406 (15.8)	3599 (20.7)	951 (16.7)
Do not use	409 (27.6)	597 (23.2)	4559 (26.2)	1654 (29.0)
Tiktok (SA)		*	*	
Almost every day	202 (13.6)	371 (14.4)	1407 (8.1)	546 (9.6)

2-5 days per week	47 (3.2)		180 (7.0)		551 (3.2)		175 (3.1)
About once a week or less	60 (4.0)		175 (6.8)		897 (5.2)		262 (4.6)
Do not use	1174 (79.2)		1847 (71.8)		14546 (83.6)		4714 (82.7)
Medical History							
Hypertension	179 (12.1)		306 (11.9)		3959 (22.8)	*	709 (12.4)
Diabetes	75 (5.1)		90 (3.5)		1313 (7.5)	*	209 (3.7)
Dyslipidemia (hyperlipidemia)	101 (6.8)		126 (4.9)		2013 (11.6)	*	272 (4.8)
Pneumonia / Bronchitis	65 (4.4)		83 (3.2)		657 (3.8)	*	155 (2.7)
Asthma	103 (6.9)	*	142 (5.5)		1016 (5.8)	*	258 (4.5)
Atopic dermatitis	90 (6.1)		168 (6.5)	*	866 (5.0)		262 (4.6)
Allergic rhinitis	132 (8.9)		220 (8.6)		1760 (10.1)	*	399 (7.0)
Periodontal disease	134 (9.0)		182 (7.1)		2293 (13.2)	*	424 (7.4)
Dental caries (cavities)	220 (14.8)		315 (12.2)		3610 (20.7)	*	841 (14.8)
Cataract	76 (5.1)		78 (3.0)		1507 (8.7)	*	221 (3.9)
Angina / Myocardial infarction	31 (2.1)		33 (1.3)		452 (2.6)	*	78 (1.4)
Stroke (cerebral infarction, cerebral hemorrhage)	16 (1.1)		24 (0.9)		245 (1.4)		52 (0.9)
COPD (Chronic Obstructive Pulmonary Disease)	9 (0.6)		7 (0.3)		71 (0.4)		13 (0.2)
Chronic kidney disease	17 (1.1)	*	11 (0.4)		161 (0.9)	*	14 (0.2)
Chronic hepatitis / Cirrhosis	12 (0.8)		19 (0.7)		104 (0.6)		18 (0.3)
Immunodeficiency or immune function decline (including those on steroids, biologics, immunosuppressants)	25 (1.7)	*	21 (0.8)		194 (1.1)	*	31 (0.5)
Cancer / Malignant tumor	51 (3.4)		75 (2.9)		1141 (6.6)	*	217 (3.8)
Chronic pain (e.g., persistent back pain, headache for over three months)	50 (3.4)		57 (2.2)		785 (4.5)	*	137 (2.4)
Depression	98 (6.6)	*	115 (4.5)		773 (4.4)		246 (4.3)
Mental illness other than depression	91 (6.1)	*	94 (3.7)		608 (3.5)		203 (3.6)
None apply	792 (53.4)		1437 (55.8)		6409 (36.8)	*	3201 (56.2)
**II. Life satisfaction and social connections**							
Good Well-being	378 (25.5)	*	432 (16.8)		4020 (23.1)	*	947 (16.6)

Social Isolation	1043 (70.3)		1859 (72.3)		10363 (59.6)	*	4063 (71.3)
**III. Habitual dietary preferences and tendencies**							
Frequency Level of Drinking Beverages							
Japanese tea (SA)		*		*		*	
Less than once per week	605 (40.8)		913 (35.5)		4062 (23.3)		1917 (33.6)
1 to 2 times per week	138 (9.3)		391 (15.2)		1911 (11.0)		603 (10.6)
3 to 4 times per week	75 (5.1)		337 (13.1)		1396 (8.0)		429 (7.5)
5 to 6 times per week	92 (6.2)		233 (9.1)		1161 (6.7)		459 (8.1)
One serving daily	201 (13.6)		292 (11.3)		2664 (15.3)		837 (14.7)
2 to 3 servings daily	209 (14.1)		257 (10.0)		4101 (23.6)		961 (16.9)
4 to 6 servings daily	85 (5.7)		102 (4.0)		1569 (9.0)		332 (5.8)
7 to 9 servings daily	23 (1.6)		26 (1.0)		326 (1.9)		80 (1.4)
More than 10 servings daily	55 (3.7)		22 (0.9)		211 (1.2)		79 (1.4)
Oolong tea (SA)		*		*			
Less than once per week	1186 (80.0)		1689 (65.6)		14033 (80.6)		4497 (78.9)
1 to 2 times per week	93 (6.3)		377 (14.7)		1456 (8.4)		469 (8.2)
3 to 4 times per week	45 (3.0)		247 (9.6)		624 (3.6)		218 (3.8)
5 to 6 times per week	31 (2.1)		118 (4.6)		336 (1.9)		156 (2.7)
One serving daily	46 (3.1)		70 (2.7)		458 (2.6)		178 (3.1)
2 to 3 servings daily	34 (2.3)		36 (1.4)		310 (1.8)		108 (1.9)
4 to 6 servings daily	15 (1.0)		17 (0.7)		105 (0.6)		41 (0.7)
7 to 9 servings daily	6 (0.4)		7 (0.3)		28 (0.2)		10 (0.2)
More than 10 servings daily	27 (1.8)		12 (0.5)		51 (0.3)		20 (0.4)
Black tea (SA)		*		*		*	
Less than once per week	1084 (73.1)		1539 (59.8)		11689 (67.2)		4019 (70.5)
1 to 2 times per week	157 (10.6)		416 (16.2)		2434 (14.0)		687 (12.1)
3 to 4 times per week	63 (4.2)		283 (11.0)		1147 (6.6)		316 (5.5)
5 to 6 times per week	40 (2.7)		142 (5.5)		564 (3.2)		198 (3.5)
One serving daily	69 (4.7)		126 (4.9)		993 (5.7)		298 (5.2)
2 to 3 servings daily	33 (2.2)		34 (1.3)		412 (2.4)		118 (2.1)
4 to 6 servings daily	8 (0.5)		16 (0.6)		84 (0.5)		29 (0.5)
7 to 9 servings daily	6 (0.4)		4 (0.2)		28 (0.2)		12 (0.2)
More than 10 servings daily	23 (1.6)		13 (0.5)		50 (0.3)		20 (0.4)

Coffee (excluding canned coffee) (SA)		*		*		*	
Less than once per week	560 (37.8)		763 (29.7)		3404 (19.6)		1683 (29.5)
1 to 2 times per week	110 (7.4)		374 (14.5)		1278 (7.3)		482 (8.5)
3 to 4 times per week	72 (4.9)		300 (11.7)		1149 (6.6)		388 (6.8)
5 to 6 times per week	91 (6.1)		273 (10.6)		1312 (7.5)		495 (8.7)
One serving daily	270 (18.2)		415 (16.1)		4351 (25.0)		1172 (20.6)
2 to 3 servings daily	241 (16.3)		340 (13.2)		4732 (27.2)		1137 (20.0)
4 to 6 servings daily	74 (5.0)		74 (2.9)		874 (5.0)		240 (4.2)
7 to 9 servings daily	21 (1.4)		17 (0.7)		186 (1.1)		45 (0.8)
More than 10 servings daily	44 (3.0)		17 (0.7)		115 (0.7)		55 (1.0)
Canned coffee (SA)		*		*		*	
Less than once per week	1164 (78.5)		1684 (65.4)		14294 (82.1)		4405 (77.3)
1 to 2 times per week	99 (6.7)		346 (13.4)		1366 (7.9)		483 (8.5)
3 to 4 times per week	51 (3.4)		231 (9.0)		602 (3.5)		255 (4.5)
5 to 6 times per week	49 (3.3)		127 (4.9)		329 (1.9)		170 (3.0)
One serving daily	64 (4.3)		116 (4.5)		514 (3.0)		249 (4.4)
2 to 3 servings daily	24 (1.6)		45 (1.7)		198 (1.1)		82 (1.4)
4 to 6 servings daily	7 (0.5)		4 (0.2)		35 (0.2)		22 (0.4)
7 to 9 servings daily	5 (0.3)		6 (0.2)		21 (0.1)		6 (0.1)
More than 10 servings daily	20 (1.3)		14 (0.5)		42 (0.2)		25 (0.4)
Importance of Choices in Meals, Ingredients, and Groceries, mean (standard deviation) [min 0, max 7]							
Convenience in daily life (e.g., quick cooking, no hassle)	4.25 (2.24)	*	3.80 (1.51)	*	4.79 (1.04)	*	4.42 (1.08)
Preference in taste (e.g., strong, light, spicy)	4.64 (2.26)		3.93 (1.57)	*	5.07 (1.00)	*	4.62 (1.12)
Familiarity due to family or culture (e.g., eaten since childhood, family's traditional dishes)	3.53 (2.27)	*	3.29 (1.38)	*	4.37 (1.14)	*	4.00 (1.08)
Budget or price	5.05 (2.14)	*	4.26 (1.67)	*	5.35 (1.05)	*	4.89 (1.24)
Quality	4.58 (2.34)		3.87 (1.50)	*	5.34 (1.01)	*	4.65 (1.12)
Safety	4.68 (2.39)		3.98 (1.60)	*	5.61 (1.06)	*	4.80 (1.20)
Manufacturer or brand	3.43 (2.16)	*	3.25 (1.32)	*	4.20 (1.15)	*	3.86 (1.07)

Food allergies or physical constitution	3.44 (2.40)	*	3.11 (1.48)	*	4.09 (1.49)	*	3.71 (1.32)
Religion or beliefs	2.26 (1.92)	*	2.44 (1.35)	*	2.81 (1.52)		2.86 (1.43)
Medical considerations (restrictions due to illness or disability)	3.43 (2.40)	*	3.08 (1.36)	*	4.38 (1.33)	*	3.75 (1.20)
Origin of ingredients	3.71 (2.34)	*	3.29 (1.40)	*	4.60 (1.19)	*	4.00 (1.13)
Considerations of health and nutrition	4.01 (2.50)	*	3.54 (1.39)	*	5.20 (1.00)	*	4.37 (1.02)
Criteria related to best before and use-by dates	4.22 (2.26)	*	3.71 (1.48)	*	4.88 (1.10)	*	4.39 (1.12)
Freshness	4.65 (2.32)		3.97 (1.54)	*	5.50 (1.01)	*	4.76 (1.14)
Method of preservation (e.g., frozen, refrigerated, ambient)	4.07 (2.33)	*	3.58 (1.44)	*	4.85 (1.05)	*	4.29 (1.07)
Respect for culture and tradition	3.07 (2.17)	*	2.97 (1.32)	*	4.03 (1.22)	*	3.64 (1.15)
Impact on the environment (e.g., sustainability, organic)	3.24 (2.32)	*	2.94 (1.28)	*	4.21 (1.17)	*	3.61 (1.12)
Seasonality	3.82 (2.36)	*	3.42 (1.40)	*	4.84 (1.10)	*	4.21 (1.08)
Background information of ingredients and dishes (e.g., information about the producer)	3.34 (2.29)	*	3.04 (1.29)	*	4.23 (1.15)	*	3.70 (1.10)
Importance of the Scene or Environment for Eating Out, mean (standard deviation) [min 0, max 7]							
Environment conducive to enjoying conversation and communication	3.86 (2.22)	*	3.53 (1.41)	*	4.72 (1.12)	*	4.17 (1.13)
Consideration of the pace of dining (e.g., slow dining, fast service)	3.99 (2.22)	*	3.60 (1.43)	*	4.71 (1.06)	*	4.20 (1.09)
Sound environment (e.g., background noise, appropriate music)	3.64 (2.16)	*	3.38 (1.42)	*	4.41 (1.15)	*	3.99 (1.11)
Introduction of natural light	3.46 (2.20)	*	3.24 (1.35)	*	4.32 (1.18)	*	3.84 (1.13)
Appropriate lighting environment	3.79 (2.17)	*	3.47 (1.37)	*	4.56 (1.07)	*	4.11 (1.06)
Temperature setting (e.g., adjusted for the season)	4.09 (2.20)	*	3.68 (1.44)	*	4.86 (1.03)	*	4.33 (1.07)

Preferred location or seating (e.g., near a window or on a terrace)	3.71 (2.18)	*	3.44 (1.41)	*	4.45 (1.12)	*	4.03 (1.09)
Comfort of seating (e.g., chairs, height of tables)	3.99 (2.19)	*	3.60 (1.44)	*	4.74 (1.07)	*	4.25 (1.09)
Unique design or theme (e.g., scenery or culture)	3.18 (2.04)	*	3.05 (1.30)	*	3.90 (1.17)	*	3.61 (1.10)
Provision of privacy (e.g., private rooms or semi-private rooms)	3.85 (2.22)	*	3.55 (1.44)	*	4.54 (1.15)	*	4.11 (1.11)
Acceptance of all family members (e.g., with children or pets)	3.58 (2.23)	*	3.35 (1.48)	*	4.34 (1.31)	*	3.98 (1.24)
Digital environment (e.g., Wi-Fi, charging facilities)	3.49 (2.19)	*	3.28 (1.46)	*	4.04 (1.41)	*	3.74 (1.30)
Privacy of conversation	3.97 (2.23)	*	3.63 (1.45)	*	4.73 (1.12)	*	4.21 (1.12)
Modern ordering system (e.g., touch panels)	3.08 (2.05)	*	3.03 (1.30)	*	3.67 (1.23)	*	3.53 (1.15)
Visual information about ingredients and dishes (e.g., photos or descriptions)	3.61 (2.13)	*	3.38 (1.37)	*	4.31 (1.14)	*	3.97 (1.10)
Frequency of Snacking or Break Time (SA)		*		*		*	
More than twice a day	193 (13.0)		212 (8.2)		2142 (12.3)		511 (9.0)
Once a day	319 (21.5)		537 (20.9)		4861 (27.9)		1198 (21.0)
4-6 times a week	102 (6.9)		305 (11.9)		1647 (9.5)		447 (7.8)
2-3 times a week	137 (9.2)		359 (14.0)		2642 (15.2)		758 (13.3)
About once a week	92 (6.2)		219 (8.5)		1302 (7.5)		408 (7.2)
1-2 times a month	48 (3.2)		121 (4.7)		678 (3.9)		230 (4.0)
Rarely	592 (39.9)		820 (31.9)		4129 (23.7)		2145 (37.7)
Main Time for Snacking or Break Time							
Morning (after breakfast until before lunch)	145 (9.8)		189 (7.3)		2155 (12.4)	*	451 (7.9)
After lunch until 3 PM	270 (18.2)		463 (18.0)		4483 (25.8)	*	1012 (17.8)
3 PM until before dinner	422 (28.5)		832 (32.3)		7134 (41.0)	*	1719 (30.2)
After dinner	214 (14.4)		418 (16.2)		2708 (15.6)		882 (15.5)
Late at night	84 (5.7)		147 (5.7)	*	516 (3.0)		216 (3.8)
No specific time	136 (9.2)		215 (8.4)		1253 (7.2)	*	514 (9.0)
Rarely	541 (36.5)		765 (29.7)	*	3465 (19.9)	*	1957 (34.4)

Selection of Foods Normally Not Chosen in Specific Situations or Contexts (SA)		*		*		*	
Often	102 (6.9)		122 (4.7)		808 (4.6)		160 (2.8)
Occasionally	263 (17.7)		622 (24.2)		5042 (29.0)		1035 (18.2)
Rarely	228 (15.4)		701 (27.2)		5291 (30.4)		1506 (26.4)
Almost never	890 (60.0)		1128 (43.8)		6260 (36.0)		2996 (52.6)
**IV. Everyday meal experiences**							
Meals Usually Prepared by Oneself	827 (55.8)		1315 (51.1)		10865 (62.4)	*	3032 (53.2)
Number of Days Eating Alone in the Past 7 Days							
Breakfast (SA)				*		*	
0 days	389 (26.2)		564 (21.9)		5422 (31.2)		1446 (25.4)
1-2 days	118 (8.0)		346 (13.4)		1847 (10.6)		577 (10.1)
3-4 days	97 (6.5)		302 (11.7)		1265 (7.3)		411 (7.2)
5-6 days	114 (7.7)		263 (10.2)		1473 (8.5)		463 (8.1)
Every day (7 days)	482 (32.5)		704 (27.4)		5891 (33.9)		1916 (33.6)
Usually do not eat breakfast	283 (19.1)		394 (15.3)		1503 (8.6)		884 (15.5)
Lunch (SA)		*		*		*	
0 days	288 (19.4)		400 (15.5)		4092 (23.5)		1114 (19.6)
1-2 days	177 (11.9)		413 (16.1)		2999 (17.2)		840 (14.7)
3-4 days	160 (10.8)		454 (17.6)		2346 (13.5)		746 (13.1)
5-6 days	191 (12.9)		420 (16.3)		2843 (16.3)		925 (16.2)
Every day (7 days)	497 (33.5)		681 (26.5)		4474 (25.7)		1697 (29.8)
Usually do not eat lunch	170 (11.5)		205 (8.0)		647 (3.7)		375 (6.6)
Dinner (SA)		*		*		*	
0 days	580 (39.1)		884 (34.4)		8982 (51.6)		2470 (43.4)
1-2 days	152 (10.2)		391 (15.2)		2162 (12.4)		658 (11.5)
3-4 days	97 (6.5)		328 (12.7)		1263 (7.3)		464 (8.1)
5-6 days	119 (8.0)		290 (11.3)		1275 (7.3)		483 (8.5)
Every day (7 days)	618 (41.7)		545 (21.2)		6497 (37.3)		1419 (24.9)
Usually do not eat dinner	106 (7.1)		91 (3.5)		203 (1.2)		154 (2.7)
Frequently Experienced Conversation Situations							
Breakfast (SA)		*		*		*	
No conversation	920 (62.0)		1324 (51.5)		7935 (45.6)		3200 (56.2)

Simple conversation: Basic greetings or essential communication only	269 (18.1)	699 (27.2)	5072 (29.1)	1382 (24.3)
Normal conversation: Everyday communication with friends or family	238 (16.0)	491 (19.1)	4184 (24.0)	1034 (18.1)
Deep/long conversation: Detailed discussion on specific themes or topics	56 (3.8)	59 (2.3)	210 (1.2)	81 (1.4)
Lunch (SA)		*	*	*
No conversation	834 (56.2)	1109 (43.1)	6568 (37.7)	2751 (48.3)
Simple conversation: Basic greetings or essential communication only	249 (16.8)	703 (27.3)	4356 (25.0)	1370 (24.0)
Normal conversation: Everyday communication with friends or family	335 (22.6)	700 (27.2)	6192 (35.6)	1471 (25.8)
Deep/long conversation: Detailed discussion on specific themes or topics	29 (2.0)	153 (5.9)	275 (1.6)	167 (2.9)
Dinner (SA)		*	*	*
No conversation	611 (41.2)	725 (28.2)	3742 (21.5)	1866 (32.8)
Simple conversation: Basic greetings or essential communication only	232 (15.6)	712 (27.7)	3180 (18.3)	1252 (22.0)
Normal conversation: Everyday communication with friends or family	432 (29.1)	948 (36.8)	5410 (31.1)	2670 (46.9)
Deep/long conversation: Detailed discussion on specific themes or topics	163 (11.0)	170 (6.6)	1549 (8.9)	327 (5.7)
Satisfaction with Meals				
Breakfast (SA)		*	*	*
Not satisfied at all	202 (13.6)	186 (7.2)	343 (2.0)	181 (3.2)
Not very satisfied	83 (5.6)	351 (13.6)	1068 (6.1)	368 (6.5)
Neither satisfied nor dissatisfied	477 (32.2)	966 (37.5)	8930 (51.3)	2252 (39.5)
Somewhat satisfied	368 (24.8)	819 (31.8)	7983 (45.9)	1849 (32.5)
Very satisfied	398 (26.8)	269 (10.5)	2597 (14.9)	629 (11.0)
Lunch (SA)		*	*	*
Not satisfied at all	177 (11.9)	115 (4.5)	231 (1.3)	138 (2.4)

Not very satisfied	97 (6.5)	413 (16.1)	960 (5.5)	382 (6.7)
Neither satisfied nor dissatisfied	411 (27.7)	915 (35.6)	5561 (32.0)	2581 (45.3)
Somewhat satisfied	408 (27.5)	872 (33.9)	8245 (47.4)	1958 (34.4)
Very satisfied	390 (26.3)	258 (10.0)	2404 (13.8)	638 (11.2)
Dinner (SA)	*	*	*	
Not satisfied at all	160 (10.8)	111 (4.3)	166 (1.0)	118 (2.1)
Not very satisfied	97 (6.5)	334 (13.0)	704 (4.0)	307 (5.4)
Neither satisfied nor dissatisfied	328 (22.1)	791 (30.7)	3384 (19.4)	2131 (37.4)
Somewhat satisfied	437 (29.5)	963 (37.4)	9001 (51.7)	2233 (39.2)
Very satisfied	476 (32.1)	389 (15.1)	4177 (24.0)	950 (16.7)
Perceived Time Availability During Meals				
Breakfast (SA)	*	*	*	
Not sufficient at all	279 (18.8)	279 (10.8)	1001 (5.8)	348 (6.1)
Not very sufficient	176 (11.9)	562 (21.8)	3124 (18.0)	862 (15.1)
Neither sufficient nor insufficient	315 (21.2)	766 (29.8)	3653 (21.0)	2163 (38.0)
Somewhat sufficient	258 (17.4)	679 (26.4)	6076 (34.9)	1508 (26.5)
Very sufficient	455 (30.7)	287 (11.2)	3547 (20.4)	816 (14.3)
Lunch (SA)	*	*	*	
Not sufficient at all	166 (11.2)	130 (5.1)	347 (2.0)	150 (2.6)
Not very sufficient	164 (11.1)	454 (17.6)	1478 (8.5)	501 (8.8)
Neither sufficient nor insufficient	315 (21.2)	776 (30.2)	4025 (23.1)	2174 (38.2)
Somewhat sufficient	346 (23.3)	833 (32.4)	7486 (43.0)	1893 (33.2)
Very sufficient	492 (33.2)	380 (14.8)	4065 (23.4)	979 (17.2)
Dinner (SA)	*	*	*	
Not sufficient at all	150 (10.1)	100 (3.9)	190 (1.1)	111 (1.9)
Not very sufficient	82 (5.5)	319 (12.4)	673 (3.9)	265 (4.7)
Neither sufficient nor insufficient	253 (17.1)	660 (25.7)	2289 (13.2)	1758 (30.9)
Somewhat sufficient	365 (24.6)	934 (36.3)	7721 (44.4)	2102 (36.9)
Very sufficient	429 (28.9)	589 (22.9)	3516 (20.2)	1468 (25.8)
Frequency of Meal Types				
Homemade meals at home (SA)	*	*	*	

Almost every day	738 (49.8)	1028 (40.0)	11235 (64.6)	2827 (49.6)
3-4 times a week	188 (12.7)	465 (18.1)	2919 (16.8)	848 (14.9)
1-2 times a week	123 (8.3)	437 (17.0)	1273 (7.3)	556 (9.8)
1-2 times a month	64 (4.3)	221 (8.6)	555 (3.2)	312 (5.5)
Once or twice every six months	41 (2.8)	145 (5.6)	497 (2.9)	194 (3.4)
Rarely/Never	322 (21.7)	311 (12.1)	1095 (6.3)	969 (17.0)
Eating out (restaurants, fast food, etc.) (SA)	*	*	*	
Almost every day	71 (4.8)	78 (3.0)	128 (0.7)	74 (1.3)
3-4 times a week	58 (3.9)	239 (9.3)	433 (2.5)	181 (3.2)
1-2 times a week	205 (13.8)	544 (21.1)	2573 (14.8)	840 (14.7)
1-2 times a month	434 (29.3)	734 (28.5)	6641 (38.2)	1789 (31.4)
Once or twice every six months	221 (14.9)	428 (16.6)	4095 (23.5)	1095 (19.2)
Rarely/Never	494 (33.3)	550 (21.4)	3531 (20.3)	1718 (30.2)
Takeout (bento, onigiri, etc.) (SA)	*	*	*	
Almost every day	108 (7.3)	117 (4.5)	255 (1.5)	151 (2.7)
3-4 times a week	93 (6.3)	297 (11.5)	760 (4.4)	295 (5.2)
1-2 times a week	166 (11.2)	514 (20.0)	2523 (14.5)	836 (14.7)
1-2 times a month	263 (17.7)	550 (21.4)	4610 (26.5)	1271 (22.3)
Once or twice every six months	186 (12.5)	353 (13.7)	3395 (19.5)	862 (15.1)
Rarely/Never	667 (45.0)	742 (28.8)	5858 (33.7)	2282 (40.1)
Delivery services (SA)	*	*	*	
Almost every day	66 (4.5)	58 (2.3)	74 (0.4)	41 (0.7)
3-4 times a week	25 (1.7)	190 (7.4)	112 (0.6)	70 (1.2)
1-2 times a week	51 (3.4)	282 (11.0)	347 (2.0)	207 (3.6)
1-2 times a month	87 (5.9)	235 (9.1)	880 (5.1)	349 (6.1)
Once or twice every six months	112 (7.6)	246 (9.6)	1718 (9.9)	480 (8.4)
Rarely/Never	1148 (77.4)	1560 (60.6)	14265 (82.0)	4546 (79.8)
Eat-in at convenience stores or shopping malls (SA)	*	*	*	
Almost every day	74 (5.0)	84 (3.3)	86 (0.5)	52 (0.9)
3-4 times a week	50 (3.4)	244 (9.5)	269 (1.5)	145 (2.5)
1-2 times a week	134 (9.0)	230 (8.9)	827 (4.8)	357 (6.3)

1-2 times a month	176 (11.9)	422 (16.4)	2519 (14.5)	804 (14.1)
Once or twice every six months	206 (13.9)	426 (16.6)	3735 (21.5)	930 (16.3)
Rarely/Never	864 (58.3)	1315 (51.1)	7027 (40.4)	3366 (59.1)
Prepared foods from supermarkets (SA)	*	*	*	
Almost every day	107 (7.2)	105 (4.1)	388 (2.2)	194 (3.4)
3-4 times a week	120 (8.1)	387 (15.0)	1581 (9.1)	500 (8.8)
1-2 times a week	319 (21.5)	744 (28.9)	5476 (31.5)	1479 (26.0)
1-2 times a month	340 (22.9)	588 (22.9)	5210 (29.9)	1378 (24.2)
Once or twice every six months	125 (8.4)	292 (11.3)	2061 (11.8)	663 (11.6)
Rarely/Never	472 (31.8)	457 (17.8)	2685 (15.4)	1483 (26.0)
Light meals at cafes or tea shops (SA)	*	*	*	
Almost every day	62 (4.2)	68 (2.6)	56 (0.3)	43 (0.8)
3-4 times a week	32 (2.2)	167 (6.5)	193 (1.1)	102 (1.8)
1-2 times a week	89 (6.0)	357 (13.9)	1001 (5.8)	366 (6.4)
1-2 times a month	206 (13.9)	402 (15.6)	3209 (18.4)	826 (14.5)
Once or twice every six months	203 (13.7)	470 (18.3)	4145 (23.8)	1025 (18.0)
Rarely/Never	886 (59.7)	1101 (42.8)	8802 (50.6)	3333 (58.5)
School or company cafeterias/canteens (SA)	*	*	*	
Almost every day	94 (6.3)	113 (4.4)	376 (2.2)	157 (2.8)
3-4 times a week	55 (3.7)	166 (6.5)	1268 (7.3)	308 (5.4)
1-2 times a week	45 (3.0)	281 (10.9)	541 (3.1)	271 (4.8)
1-2 times a month	35 (2.4)	208 (8.1)	486 (2.8)	239 (4.2)
Once or twice every six months	45 (3.0)	135 (5.2)	552 (3.2)	211 (3.7)
Rarely/Never	1198 (80.8)	1578 (61.3)	14839 (85.3)	4589 (80.6)
Instant food (SA)	*	*	*	
Almost every day	103 (6.9)	98 (3.8)	211 (1.2)	148 (2.6)
3-4 times a week	91 (6.1)	331 (12.9)	796 (4.6)	365 (6.4)
1-2 times a week	292 (19.7)	702 (27.3)	4330 (24.9)	1383 (24.3)
1-2 times a month	359 (24.2)	672 (26.1)	6271 (36.0)	1706 (29.9)
Once or twice every six months	155 (10.5)	320 (12.4)	2742 (15.8)	751 (13.2)

Rarely/Never	483 (32.6)	450 (17.5)	3051 (17.5)	1344 (23.6)
Frozen food (SA)	*	*	*	
Almost every day	116 (7.8)	107 (4.2)	408 (2.3)	193 (3.4)
3-4 times a week	144 (9.7)	345 (13.4)	1600 (9.2)	525 (9.2)
1-2 times a week	292 (19.7)	711 (27.6)	5227 (30.0)	1435 (25.2)
1-2 times a month	315 (21.2)	586 (22.8)	5243 (30.1)	1376 (24.2)
Once or twice every six months	138 (9.3)	310 (12.0)	2126 (12.2)	653 (11.5)
Rarely/Never	478 (32.2)	514 (20.0)	2797 (16.1)	1515 (26.6)
Frequency of Methods for Purchasing Ingredients and Groceries				
Supermarket (SA)	*	*	*	
Almost every day	248 (16.7)	271 (10.5)	2080 (12.0)	594 (10.4)
3-4 times a week	292 (19.7)	654 (25.4)	5231 (30.1)	1262 (22.2)
1-2 times a week	544 (36.7)	1044 (40.6)	8104 (46.6)	2395 (42.0)
1-2 times a month	153 (10.3)	300 (11.7)	1213 (7.0)	590 (10.4)
Once or twice every six months	35 (2.4)	103 (4.0)	307 (1.8)	192 (3.4)
Rarely/Never	211 (14.2)	201 (7.8)	466 (2.7)	664 (11.7)
Convenience store (SA)	*	*	*	
Almost every day	91 (6.1)	92 (3.6)	347 (2.0)	201 (3.5)
3-4 times a week	103 (6.9)	277 (10.8)	843 (4.8)	329 (5.8)
1-2 times a week	217 (14.6)	587 (22.8)	2663 (15.3)	917 (16.1)
1-2 times a month	247 (16.7)	526 (20.4)	4253 (24.4)	1171 (20.6)
Once or twice every six months	167 (11.3)	370 (14.4)	3203 (18.4)	828 (14.5)
Rarely/Never	660 (44.5)	699 (27.2)	6181 (35.5)	2312 (40.6)
Local market or direct sales outlet (SA)	*	*	*	
Almost every day	67 (4.5)	76 (3.0)	51 (0.3)	45 (0.8)
3-4 times a week	37 (2.5)	205 (8.0)	344 (2.0)	96 (1.7)
1-2 times a week	105 (7.1)	372 (14.5)	1587 (9.1)	420 (7.4)
1-2 times a month	158 (10.7)	348 (13.5)	2908 (16.7)	649 (11.4)
Once or twice every six months	168 (11.3)	354 (13.8)	3621 (20.8)	837 (14.7)
Rarely/Never	949 (64.0)	1236 (48.0)	8867 (51.0)	3654 (64.1)

Specialty stores (butcher, fishmonger, greengrocer, etc.) (SA)	*	*	*	
Almost every day	70 (4.7)	65 (2.5)	83 (0.5)	46 (0.8)
3-4 times a week	47 (3.2)	202 (7.9)	436 (2.5)	140 (2.5)
1-2 times a week	103 (6.9)	387 (15.0)	1806 (10.4)	487 (8.5)
1-2 times a month	142 (9.6)	356 (13.8)	2745 (15.8)	683 (12.0)
Once or twice every six months	145 (9.8)	301 (11.7)	2883 (16.6)	665 (11.7)
Rarely/Never	976 (65.8)	1262 (49.0)	9448 (54.3)	3676 (64.5)
Online shop (SA)	*	*	*	
Almost every day	52 (3.5)	48 (1.9)	49 (0.3)	33 (0.6)
3-4 times a week	17 (1.1)	165 (6.4)	63 (0.4)	40 (0.7)
1-2 times a week	74 (5.0)	289 (11.2)	674 (3.9)	255 (4.5)
1-2 times a month	148 (10.0)	306 (11.9)	2117 (12.2)	565 (9.9)
Once or twice every six months	158 (10.7)	310 (12.0)	2912 (16.7)	731 (12.8)
Rarely/Never	1016 (68.5)	1445 (56.2)	11564 (66.5)	4041 (70.9)
Meal kits (SA)	*	*	*	
Almost every day	54 (3.6)	46 (1.8)	29 (0.2)	31 (0.5)
3-4 times a week	27 (1.8)	153 (5.9)	106 (0.6)	61 (1.1)
1-2 times a week	22 (1.5)	199 (7.7)	119 (0.7)	113 (2.0)
1-2 times a month	62 (4.2)	225 (8.7)	637 (3.7)	263 (4.6)
Once or twice every six months	57 (3.8)	160 (6.2)	1190 (6.8)	282 (4.9)
Rarely/Never	1243 (83.8)	1645 (63.9)	15259 (87.7)	4851 (85.2)
Direct purchase from farmers or producers (SA)	*	*	*	
Almost every day	53 (3.6)	48 (1.9)	31 (0.2)	27 (0.5)
3-4 times a week	36 (2.4)	186 (7.2)	187 (1.1)	64 (1.1)
1-2 times a week	65 (4.4)	313 (12.2)	872 (5.0)	278 (4.9)
1-2 times a month	98 (6.6)	263 (10.2)	1768 (10.2)	426 (7.5)
Once or twice every six months	120 (8.1)	249 (9.7)	2429 (14.0)	524 (9.2)
Rarely/Never	1113 (75.1)	1512 (58.8)	12097 (69.5)	4371 (76.7)
Organic or pesticide-free specialty stores or online shops (SA)	*	*	*	

Almost every day	56 (3.8)	49 (1.9)	35 (0.2)	35 (0.6)
3-4 times a week	33 (2.2)	183 (7.1)	129 (0.7)	73 (1.3)
1-2 times a week	61 (4.1)	281 (10.9)	428 (2.5)	179 (3.1)
1-2 times a month	62 (4.2)	211 (8.2)	723 (4.2)	271 (4.8)
Once or twice every six months	47 (3.2)	195 (7.6)	906 (5.2)	294 (5.2)
Rarely/Never	1224 (82.5)	1685 (65.5)	14919 (85.7)	4864 (85.4)
Large discount stores (SA)	*	*	*	
Almost every day	51 (3.4)	39 (1.5)	28 (0.2)	23 (0.4)
3-4 times a week	32 (2.2)	178 (6.9)	165 (0.9)	77 (1.4)
1-2 times a week	55 (3.7)	325 (12.6)	641 (3.7)	290 (5.1)
1-2 times a month	99 (6.7)	309 (12.0)	1637 (9.4)	520 (9.1)
Once or twice every six months	159 (10.7)	319 (12.4)	2759 (15.9)	698 (12.3)
Rarely/Never	1075 (72.5)	1377 (53.5)	12150 (69.8)	4080 (71.6)
Most Common Mode of Transportation for Grocery Shopping (SA)	*	*	*	
Walking	466 (31.4)	654 (25.4)	4494 (25.8)	1632 (28.6)
Bicycle	263 (17.7)	454 (17.6)	2790 (16.0)	972 (17.1)
Self-driven car or motorcycle	552 (37.2)	1143 (44.4)	8288 (47.6)	2518 (44.2)
Car or motorcycle driven by someone else	138 (9.3)	229 (8.9)	1506 (8.7)	432 (7.6)
Bus, taxi, train	56 (3.8)	85 (3.3)	268 (1.5)	115 (2.0)
Other	8 (0.5)	8 (0.3)	55 (0.3)	28 (0.5)
Frequency of Participation in Social Dining or Food-centered Events				
Office or school lunch gatherings (SA)	*	*	*	
Almost every day	50 (3.4)	45 (1.7)	48 (0.3)	27 (0.5)
3-4 times a week	23 (1.6)	182 (7.1)	135 (0.8)	72 (1.3)
1-2 times a week	26 (1.8)	220 (8.6)	201 (1.2)	129 (2.3)
1-2 times a month	39 (2.6)	189 (7.3)	493 (2.8)	204 (3.6)
Once or twice every six months	83 (5.6)	189 (7.3)	1032 (5.9)	295 (5.2)
Rarely/Never	1244 (83.9)	1710 (66.5)	14733 (84.7)	4885 (85.7)

Dining out with friends or colleagues (SA)		*		*		*	
Almost every day	45 (3.0)		46 (1.8)		29 (0.2)		25 (0.4)
3-4 times a week	18 (1.2)		104 (4.0)		275 (1.6)		78 (1.4)
1-2 times a week	70 (4.7)		322 (12.5)		802 (4.6)		295 (5.2)
1-2 times a month	214 (14.4)		454 (17.6)		3648 (21.0)		936 (16.4)
Once or twice every six months	283 (19.1)		548 (21.3)		5829 (33.5)		1296 (22.7)
Rarely/Never	836 (56.4)		1052 (40.9)		6944 (39.9)		3089 (54.2)
Special occasion meals with family or friends (birthdays, weddings, etc.) (SA)		*		*		*	
Almost every day	52 (3.5)		49 (1.9)		33 (0.2)		26 (0.5)
3-4 times a week	19 (1.3)		149 (5.8)		51 (0.3)		52 (0.9)
1-2 times a week	27 (1.8)		241 (9.4)		173 (1.0)		136 (2.4)
1-2 times a month	92 (6.2)		263 (10.2)		1365 (7.8)		396 (7.0)
Once or twice every six months	425 (28.7)		754 (29.3)		8445 (48.5)		1843 (32.4)
Rarely/Never	868 (58.5)		1117 (43.4)		7334 (42.1)		3244 (56.9)
Outdoor dining like barbecues or picnics (SA)		*		*		*	
Almost every day	42 (2.8)		48 (1.9)		24 (0.1)		23 (0.4)
3-4 times a week	17 (1.1)		174 (6.8)		51 (0.3)		37 (0.6)
1-2 times a week	24 (1.6)		223 (8.7)		141 (0.8)		119 (2.1)
1-2 times a month	35 (2.4)		158 (6.1)		380 (2.2)		175 (3.1)
Once or twice every six months	167 (11.3)		347 (13.5)		3174 (18.2)		712 (12.5)
Rarely/Never	1193 (80.4)		1629 (63.3)		13647 (78.4)		4618 (81.1)
Online meal gatherings (SA)		*		*		*	
Almost every day	51 (3.4)		50 (1.9)		48 (0.3)		34 (0.6)
3-4 times a week	20 (1.3)		142 (5.5)		56 (0.3)		48 (0.8)
1-2 times a week	38 (2.6)		244 (9.5)		229 (1.3)		125 (2.2)
1-2 times a month	65 (4.4)		61 (2.4)		285 (1.6)		105 (1.8)
Once or twice every six months	48 (3.2)		111 (4.3)		324 (1.9)		185 (3.2)
Rarely/Never	1326 (89.4)		1841 (71.6)		16410 (94.3)		5164 (90.6)
Cooking classes or food workshops (SA)		*		*		*	

Almost every day	46 (3.1)	47 (1.8)	21 (0.1)	27 (0.5)
3-4 times a week	16 (1.1)	154 (6.0)	56 (0.3)	38 (0.7)
1-2 times a week	30 (2.0)	212 (8.2)	117 (0.7)	104 (1.8)
1-2 times a month	27 (1.8)	180 (7.0)	300 (1.7)	161 (2.8)
Once or twice every six months	51 (3.4)	146 (5.7)	532 (3.1)	203 (3.6)
Rarely/Never	1309 (88.3)	1846 (71.7)	16375 (94.1)	5154 (90.5)
Casual meetings or study groups in cafes or tea shops (SA)	*	*	*	
Almost every day	51 (3.4)	57 (2.2)	22 (0.1)	28 (0.5)
3-4 times a week	20 (1.3)	138 (5.4)	67 (0.4)	50 (0.9)
1-2 times a week	17 (1.1)	221 (8.6)	125 (0.7)	105 (1.8)
1-2 times a month	51 (3.4)	188 (7.3)	776 (4.5)	243 (4.3)
Once or twice every six months	79 (5.3)	236 (9.2)	1574 (9.0)	366 (6.4)
Rarely/Never	1258 (84.8)	1777 (69.1)	15521 (89.2)	4981 (87.4)
Catered meals at events or parties (SA)	*	*	*	
Almost every day	63 (4.2)	65 (2.5)	49 (0.3)	32 (0.6)
3-4 times a week	26 (1.8)	156 (6.1)	48 (0.3)	43 (0.8)
1-2 times a week	46 (3.1)	277 (10.8)	421 (2.4)	183 (3.2)
1-2 times a month	27 (1.8)	184 (7.2)	311 (1.8)	185 (3.2)
Once or twice every six months	87 (5.9)	213 (8.3)	1279 (7.4)	319 (5.6)
Rarely/Never	1279 (86.2)	1773 (68.9)	15620 (89.8)	5014 (88.0)
After-work events with meals or drinks (SA)	*	*	*	
Almost every day	50 (3.4)	42 (1.6)	25 (0.1)	26 (0.5)
3-4 times a week	24 (1.6)	156 (6.1)	65 (0.4)	56 (1.0)
1-2 times a week	24 (1.6)	261 (10.1)	248 (1.4)	149 (2.6)
1-2 times a month	66 (4.5)	225 (8.7)	1140 (6.6)	343 (6.0)
Once or twice every six months	144 (9.7)	315 (12.2)	2890 (16.6)	698 (12.3)
Rarely/Never	1180 (79.6)	1574 (61.2)	13032 (74.9)	4428 (77.7)
Community or local food events (SA)	*	*	*	
Almost every day	45 (3.0)	42 (1.6)	26 (0.1)	23 (0.4)
3-4 times a week	31 (2.1)	174 (6.8)	99 (0.6)	70 (1.2)

1-2 times a week	24 (1.6)		121 (4.7)		283 (1.6)		85 (1.5)
1-2 times a month	35 (2.4)		180 (7.0)		391 (2.2)		177 (3.1)
Once or twice every six months	92 (6.2)		222 (8.6)		1613 (9.3)		402 (7.1)
Rarely/Never	1266 (85.4)		1752 (68.1)		15175 (87.2)		4935 (86.6)
Use of Technology or Apps Related to Food and Ingredients							
Recipe suggestion apps (e.g., Cookpad, Delish Kitchen, etc.)	249 (16.8)		356 (13.8)		4276 (24.6)	*	765 (13.4)
Calorie and nutrition management apps (e.g., MyFitnessPal, AskKen, etc.)	67 (4.5)	*	92 (3.6)	*	600 (3.4)	*	106 (1.9)
Inventory and expiration date management apps for ingredients and groceries	52 (3.5)	*	98 (3.8)	*	356 (2.0)	*	64 (1.1)
Food waste prevention apps (e.g., notifications about ingredients and groceries nearing expiration, discounted food notifications)	56 (3.8)	*	115 (4.5)	*	388 (2.2)	*	67 (1.2)
Delivery service apps for ingredients (e.g., Oisix, Rakuten Mart, etc.)	66 (4.5)	*	92 (3.6)	*	492 (2.8)	*	88 (1.5)
Restaurant reservation and review apps (e.g., Tabelog, Retty, etc.)	206 (13.9)		361 (14.0)		3763 (21.6)	*	661 (11.6)
Apps providing information on the origin and sustainability of ingredients (e.g., CodeCheck, etc.)	51 (3.4)	*	83 (3.2)	*	299 (1.7)	*	53 (0.9)
Diet support apps for avoiding allergies or specific ingredients	34 (2.3)	*	85 (3.3)	*	230 (1.3)		54 (0.9)
Control and management apps for smart kitchen devices (e.g., IoT refrigerators, smart ovens, etc.)	48 (3.2)	*	96 (3.7)	*	265 (1.5)		74 (1.3)
AR (Augmented Reality) cooking support apps	27 (1.8)	*	71 (2.8)	*	140 (0.8)		27 (0.5)
Apps for purchasing regional specialties (e.g., selecting return gifts for Furusato	81 (5.5)	*	140 (5.4)	*	795 (4.6)	*	136 (2.4)

Nozei, specialty product market apps, etc.)								
Apps for learning new recipes or improving cooking skills (e.g., Udemy, YouTube channels, etc.)	121 (8.2)	*	203 (7.9)	*	1708 (9.8)	*	257 (4.5)
Map apps (e.g., Google Maps, etc.)	266 (17.9)	*	375 (14.6)		4287 (24.6)	*	775 (13.6)
None apply	933 (62.9)	*	1359 (52.8)	*	8600 (49.4)	*	3864 (67.8)
Other	2 (0.1)		0 (0.0)		19 (0.1)		7 (0.1)
Changes in Your Food and Ingredients-Related Habits After the COVID-19 Pandemic							
Emphasizing safety and hygiene management in choosing ingredients and groceries	183 (12.3)	*	169 (6.6)		2585 (14.9)	*	268 (4.7)
Decrease in frequency of dining out and increase in frequency of cooking at home	220 (14.8)	*	292 (11.3)		4054 (23.3)	*	579 (10.2)
Increase in use of online shopping and delivery services for ingredients and groceries	91 (6.1)	*	132 (5.1)	*	1002 (5.8)	*	162 (2.8)
Stockpiling long-lasting food items (canned goods, frozen foods, etc.)	176 (11.9)	*	216 (8.4)		3092 (17.8)	*	459 (8.1)
Interest in home gardening or urban agriculture and starting to practice it	91 (6.1)	*	121 (4.7)	*	1026 (5.9)	*	123 (2.2)
Increased awareness of supporting and purchasing locally produced ingredients and groceries	146 (9.8)	*	149 (5.8)	*	1822 (10.5)	*	187 (3.3)
Concern about the hygiene management and crowding of restaurants when dining out	173 (11.7)	*	213 (8.3)		3256 (18.7)	*	427 (7.5)
Increased interest in ingredients and groceries or supplements that boost immunity	174 (11.7)	*	159 (6.2)		2626 (15.1)	*	291 (5.1)
Gathering and learning information about the	71 (4.8)	*	98 (3.8)	*	593 (3.4)	*	76 (1.3)

supply chain and distribution of food and groceries							
Increased participation and interest in online cooking classes and meal gatherings	44 (3.0)	*	86 (3.3)	*	232 (1.3)		58 (1.0)
Efforts to reduce food waste and increase awareness of reuse	187 (12.6)	*	223 (8.7)		3042 (17.5)	*	381 (6.7)
Preference for restaurants and cafes that introduce contactless payment and automated serving	66 (4.5)	*	130 (5.1)	*	616 (3.5)	*	144 (2.5)
Interest in recipes that shorten cooking time and simple cooking foods	133 (9.0)	*	187 (7.3)		1884 (10.8)	*	302 (5.3)
Spending more time and effort on cooking, trying new recipes, and authentic dishes	100 (6.7)	*	137 (5.3)	*	978 (5.6)	*	155 (2.7)
None apply	919 (62.0)	*	1407 (54.7)	*	7360 (42.3)	*	3867 (67.9)
Other	4 (0.3)		1 (0.0)		17 (0.1)		5 (0.1)

SA refers to single-answer questions. The asterisk (*) indicates that the test result is statistically significant when comparing with reference Cluster D; the Bonferroni correction was applied for multiple testing as needed.
